# A deep‐sea sulfate‐reducing bacterium generates zero‐valent sulfur via metabolizing thiosulfate

**DOI:** 10.1002/mlf2.12038

**Published:** 2022-09-23

**Authors:** Rui Liu, Yeqi Shan, Shichuan Xi, Xin Zhang, Chaomin Sun

**Affiliations:** ^1^ CAS and Shandong Province Key Laboratory of Experimental Marine Biology Center of Deep Sea Research, Institute of Oceanology, Chinese Academy of Sciences Qingdao China; ^2^ Laboratory for Marine Biology and Biotechnology, Qingdao National Laboratory for Marine Science and Technology Qingdao China; ^3^ Center of Ocean Mega‐Science, Chinese Academy of Sciences Qingdao China; ^4^ College of Earth Science, University of Chinese Academy of Sciences Beijing China; ^5^ CAS Key Laboratory of Marine Geology and Environment Center of Deep Sea Research, Institute of Oceanology, Chinese Academy of Sciences Qingdao China; ^6^ Miami University USA

**Keywords:** cold seep, in situ, sulfate reducing bacteria, sulfide oxidation, zero‐valent sulfur

## Abstract

Zero‐valent sulfur (ZVS) is a crucial intermediate in the sulfur geobiochemical circulation and is widespread in deep‐sea cold seeps. Sulfur‐oxidizing bacteria are thought to be the major contributors to the formation of ZVS. However, ZVS production mediated by sulfate‐reducing bacteria (SRB) has rarely been reported. In this study, we isolated and cultured a typical SRB designated *Oceanidesulfovibrio marinus* CS1 from deep‐sea cold seep sediment in the South China Sea. We show that *O. marinus* CS1 forms ZVS in the medium supplemented with thiosulfate. Proteomic and protein activity assays revealed that thiosulfate reductase (PhsA) and the sulfide:quinone oxidoreductase (SQR) played key roles in driving ZVS formation in *O. marinus* CS1. During this process, thiosulfate firstly was reduced by PhsA to form sulfide, then sulfide was oxidized by SQR to produce ZVS. The expressions of PhsA and SQR were significantly upregulated when *O. marinus* CS1 was cultured in a deep‐sea cold seep, strongly indicating that strain CS1 might form ZVS in the deep‐sea environment. Notably, homologs of *phsA* and *sqr* were widely identified from microbes living in sediments of deep‐sea cold seep in the South China Sea by the metagenomic analysis. We thus propose that SRB containing *phsA* and *sqr* genes potentially contribute to the formation of ZVS in deep‐sea cold seep environments.

## Impact statement

In this study, we report a novel zero‐valent sulfur (ZVS) production pathway in a deep‐sea sulfate‐reducing bacterium (SRB) via thiosulfate metabolism. Through genome, proteome, and in vitro protein function verification and other experiments, we have confirmed that this ZVS‐producing process is mainly comprised of thiosulfate reduction by PhsA and sulfide oxidation by quinone oxidoreductase. ZVS is abundant in deep‐sea cold seep ecosystems, which has been reported to be mainly produced by sulfur‐oxidizing bacteria. However, the role of SRB in the production of ZVS has been overlooked. Our findings are important for explaining the formation of ZVS in deep‐sea cold seep sediment.

## INTRODUCTION

Zero‐valent sulfur (ZVS) is a central intermediate in the biogeochemical sulfur cycle^1^, ^3^, accumulated at sediment surfaces under the sea floor (including in cold seeps and hydrothermal vents) by both abiotic reactions and microbial metabolism^1^, ^4^, ^5^, ^6^, ^7^. In marine environments, ZVS commonly occurs in forms, such as polysulfides (S_*n*_^2−^), polymeric sulfur (S_*n*_), or cyclooctasulfur (S_8_)^8^, ^9^. Production of ZVS as the intermediate has been regarded as a biosignature of sulfide‐oxidizing microorganisms^10^, ^11^. The process of ZVS production begins with the formation of polysulfide through the oxidation of thiosulfate or sulfide^3^, ^12^, ^13^, ^14^. Formation of ZVS mediated by thiosulfate oxidation in sulfide‐oxidizing bacteria (SOB) occurs by at least four pathways^12^, ^14^, including the Sox pathway^15^, tetrathionate (S_4_I) intermediate pathway^16^,  Sox–S_4_I interaction system^17^, and a novel pathway mediated by thiosulfate dehydrogenase and thiosulfohydrolase (SoxB)^18^. For the formation of ZVS mediated by sulfide oxidation, sulfide:quinone oxidoreductase (SQR) has been proposed to be the key enzyme that catalyzes the formation of ZVS in various sulfur‐oxidizing *Alphaproteobacteria* and *Gammaproteobacteria* ^12^, ^13^, ^19^. SQR is a membrane‐associated protein that oxidizes sulfide to ZVS and transfers electrons to the membrane quinone pool with flavin adenine dinucleotide^20^. As a key sulfide detoxifying enzyme, SQR is present in many bacteria, archaea, and the mitochondria of eukaryotic cells, and is classified into six types (type I–VI)^20^, ^22^. In addition, there are many genes encoding SQR (*sqr*) identified from SOB like *Desulfurivibrio alkaliphilus* in *Deltaproteobacteria* class^23^. In fact, SQR homolog also can be found in genomes of many sulfate‐reducing bacteria (SRB; belonging to *Deltaproteobacteria*) from *Desulfobacterales* order, but their functions have not been studied because SRB remain largely uncultured^24^, ^25^.

To date, many studies have reported ZVS as an intermediate of sulfide oxidation in *Deltaproteobacteria*, but these bacteria are mainly SOB because they cannot use sulfate as the electron acceptor for growth^23^, ^26^, ^27^. Recently, a pathway of ZVS generation mediated by dissimilatory sulfate reduction has been observed in a syntrophic consortium of anaerobic methanotrophic archaea (ANME) and SRB^28^, ^29^, ^30^. In this process, ANME was proposed to drive the formation of ZVS via coupling anaerobic methane oxidation (AOM) with sulfate reduction^29^, confirming the ZVS generation from dissimilatory sulfate reduction for the first time. However, this proposal has been challenged by the suggestion that the passage of sulfur species by ANME as metabolic intermediates for their SRB partners is unlikely^31^. In addition, based on both methanogenic bioreactor and metagenomics approaches, another ZVS formation pathway mediated by dissimilatory sulfate reduction has been reported^28^, ^32^ in which SRB might utilize sulfate‐to‐ZVS as an alternative pathway to sulfate‐to‐sulfide to alleviate the inhibitive effects of sulfide. This may also need further verification given that the typical pathway of the dissimilatory sulfate reduction mediated by SRB reduces sulfate to sulfide without the production of ZVS^33^, ^34^. However, no pure cultured SRB has been isolated from the AOM enrichment cultures or methanogenic bioreactor as mentioned above^29^, ^32^. Therefore, whether SRB directly drive ZVS formation via dissimilatory sulfate reduction or other pathways is still unknown.

A typical dissimilatory sulfate reduction system contains a combination of sulfate adenylyltransferase (Sat) and adenylyl‐sulfate reductases (AprA and AprB) initiating the reduction of sulfate to sulfite, and then sulfite reductases (such as DsrA and DsrB) catalyze the reduction of sulfite to sulfide^35^. On the other hand, sulfide could also be produced by thiosulfate reduction or disproportionation in SRB^36^. Potentially, SRB might form ZVS or even elemental sulfur from sulfide, driven by SQR or other proteins with similar functions. However, to the best of our knowledge, there is no evidence indicating that a pure culture of SRB could produce ZVS as a metabolite via sulfide oxidation associated with dissimilatory sulfate reduction or thiosulfate reduction.

In the present study, a strictly anaerobic strain of *Oceanidesulfovibrio marinus* CS1 was isolated from the surface sediments of a cold seep in the South China Sea. Surprisingly, this strain of *O*. *marinus* forms ZVS in the presence of thiosulfate. With a combination of genomic, proteomic, and biochemical approaches, we demonstrate that PhsA (main subunit of thiosulfate reductase) and SQR are responsible for the formation of ZVS in strain CS1, which may be an overlooked pathway driving ZVS formation in typical SRB. Based on metagenomics analysis, the distribution of key functional genes in this pathway and its potential contribution to the deep‐sea sulfur cycle is also investigated and discussed.

## RESULTS

### Cultivation and identification of a typical sulfate‐reducing bacterium *O. marinus* CS1 from a deep‐sea cold seep

**Figure 1:**
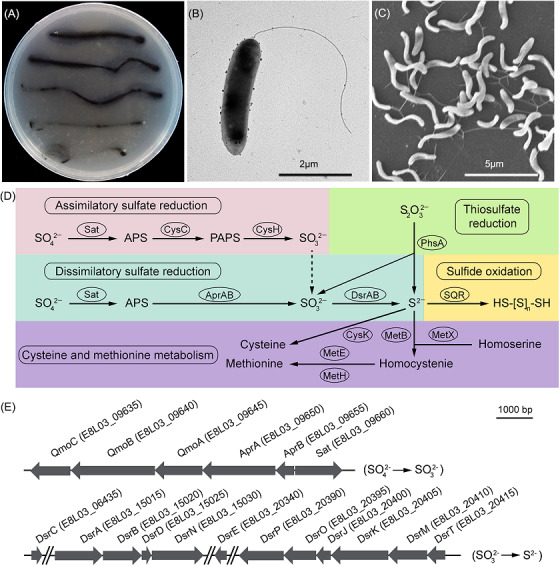
Cell morphology and genomic analysis of *Oceanidesulfovibrio marinus* CS1. (A) *O*. *marinus* CS1 forms black colonies in a solid medium containing Fe^2+^, under anaerobic conditions. (B) TEM micrograph of *O*. *marinus* CS1. (C) SEM micrograph of *O*. *marinus* CS1. (D) Proposed pathways of assimilatory sulfate reduction, dissimilatory sulfate reduction, thiosulfate reduction and sulfide oxidation, and their corelationship in *O*. *marinus* CS1. (E) Arrangement of gene clusters responsible for the reduction of sulfate to sulfite and then to sulfide. The number in parentheses after the protein name indicates its corresponding accession number. More detailed information about the proteins shown in this figure is listed in Supporting Information: Dataset Sheet S2. AprA, adenylyl‐sulfate reductase subunit alpha; AprB, adenylyl‐sulfate reductase subunit beta; APS, adenosine 5ʹ‐phosphosulfate; CysC, adenylyl‐sulfate kinase; CysH, phosphoadenosine 5ʹ‐phosphosulfate reductase family protein; DsrA, dissimilatory‐type sulfite reductase subunit alpha; DsrB, dissimilatory‐type sulfite reductase subunit beta; DsrC, TusE/DsrC/DsvC family sulfur relay protein; DsrD, dissimilatory sulfite reductase‐associated protein; DsrE, DsrE family protein; DsrJ, sulfate reduction electron transfer complex DsrMKJOP subunit; DsrK, (hereto) disulfide reductase; DsrM, sulfate reduction electron transfer complex DsrMKJOP subunit; DsrN, cobyrinate a,c‐diamide synthase; DsrO, 4Fe–4S dicluster domain‐containing protein; DsrP, polysulfide reductase; DsrT, dissimilatory sulfite reductase system component; PAPS, 3ʹ‐phosphoadenosine‐5ʹ‐phosphosulfate (3ʹ‐phosphoadenylylsulfate); PhsA, thiosulfate reductase; QmoA, CoB‐CoM heterodisulfide reductase iron‐sulfur subunit A family protein; QmoB, hydrogenase iron‐sulfur subunit; QmoC, quinone‐interacting membrane‐bound oxidoreductase complex subunit QmoC; Sat, sulfate adenylyltransferase; SEM, scanning electron microscopy; SQR, sulfide:quinone oxidoreductase; TEM, transmission electron microscopy.

Although various SRB have been reported in deep‐sea cold seeps^37^, ^38^, ^40^, a lack of cultured representatives from the deep sea has hampered a more detailed exploration of this important group. Consequently, we anaerobically enriched surface sediment samples (depth 0–20 cm in sediment) collected from a deep‐sea cold seep with a modified sulfate‐reducing medium (SRM) at 28°C for 1 month. Enriched samples were then plated on solid SRM in Hungate tubes. Given the presence of Fe^2+^ in the medium, the growth of typical SRB would be indicated as black precipitation by a chemical reaction with sulfide. As expected, the enriched samples formed a large number of black color colonies in solid SRM, indicating the dominant presence of SRB in the enrichment. Single colonies with black color were subsequently purified several times using the dilution‐to‐extinction technique at 28°C, under strictly anaerobic conditions. Based on 16S ribosomal RNA (rRNA) gene sequencing, these colonies were all identified as the same SRB strain designated CS1. The strain CS1 clustered with *O*. *marinus* E‐2^T^ (accession no. NR_043757.1) according to the 16S rRNA gene sequence phylogenetic analysis (Figure S1A). Additionally, comparative genomic relatedness analysis by average nucleotide identity (ANI) and average amino acid identity (AAI) showed that strain CS1 has 98.95% and 99.87% (Figure S1B,C) similarity respectively to *O. marinus* P48SEP (accession no. ASM762508). This is above the required threshold (95%) to designate novel species^41^, ^42^. Thus, strain CS1 is a species of *O. marinus* and is named *O. marinus* CS1 in this study. The strain CS1 showed a black color when grown on agar plates containing Fe^2+^, as expected (Figure 1A). Transmission electron microscopy (TEM) and scanning electron microscopy (SEM) showed that strain CS1 cells were approximately 2 × 0.5 μm in size, and therefore short and rod‐like in shape, with a single flagellum (Figure 1B,C).

### Diverse sulfur metabolic pathways exist in *O. marinus* CS1

To obtain deeper insights into *O. marinus* CS1, the full genome of the strain was sequenced (Supporting Information: Dataset Sheet S1). When analyzing the genome sequence of strain CS1, we found a complete dissimilatory sulfate reduction pathway and a partial assimilatory sulfate reduction pathway (Figure 1D and Supporting Information: Dataset Sheet S2). For the dissimilatory sulfate reduction pathway, two conserved gene clusters responsible for transforming sulfate to sulfite and sulfite to sulfide (Figure 1E) were identified. Surprisingly, a homologous gene encoding SQR—usually involved in sulfide oxidation—was also present in the genome of *O*. *marinus* CS1 (Figure 1D and Supporting Information: Dataset Sheet S2). Given the presence of this SQR‐like protein and its usual involvement in sulfur oxidation, we speculated that some unexplored sulfur oxidation pathways might exist in strain CS1.

### The response of *O*. *marinus* CS1 to different sulfur sources

**Figure 2:**
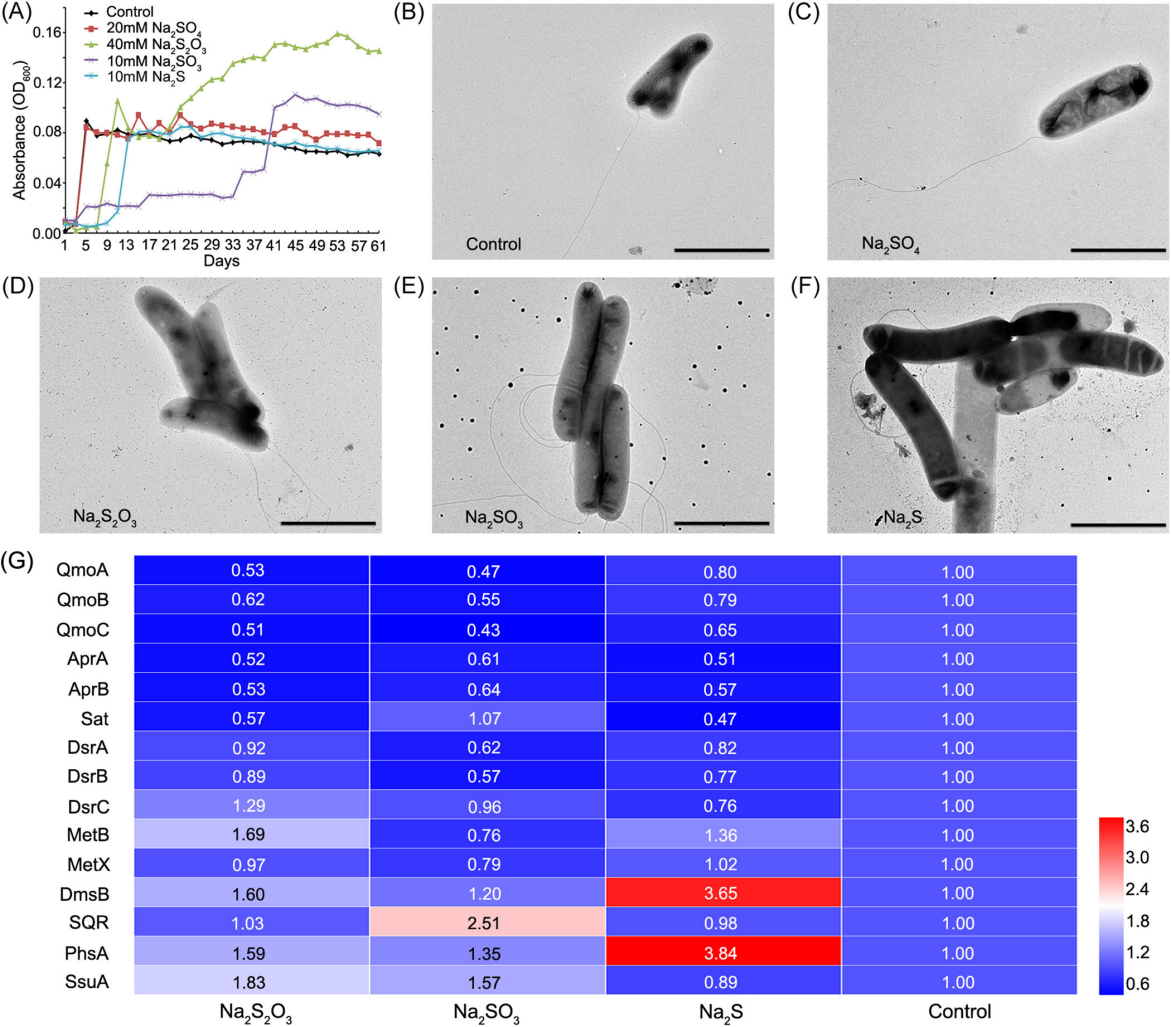
Response of *Oceanidesulfovibrio marinus* CS1 to different sulfur sources. (A) Growth of *O*. *marinus* CS1 cultured in SRM medium supplemented with different sulfur sources, including Na_2_SO_4_ (20 mM), Na_2_S_2_O_3_ (40 mM), Na_2_SO_3_ (10 mM), and Na_2_S (10 mM). “Control” indicates *O*. *marinus* CS1 cultured in SRM medium. (B–F) TEM micrographs of *O*. *marinus* CS1, when cultured in medium supplemented with different sulfur sources as shown in Panel (A). Scale bars = 2 μm. (G) Proteomics‐based heatmap, showing all significantly downregulated or upregulated proteins associated with sulfur metabolism in *O*. *marinus* CS1 when cultured in a medium supplemented with 40 mM Na_2_S_2_O_3_ 10 mM Na_2_SO_3_ or 10 mM Na_2_S, respectively. The numbers shown in the heatmap represent the fold change of proteins compared to the control group. Other abbreviations are the same as shown in Figure 1. More detailed information about the protein expression levels shown in this figure are listed in Supporting Information: Dataset Sheets S2 and S3. DmsB, anaerobic dimethyl sulfoxide reductase subunit B; MetB, cystathionine gamma‐synthase family protein; MetX, homoserine *O*‐acetyltransferase; SRM, sulfate‐reducing medium; SsuA, aliphatic sulfonate ABC transporter.

Considering the presence of diverse sulfur metabolic pathways in *O. marinus* CS1, we aimed at exploring its response to different sulfur‐containing compounds, including sulfate, sulfite, thiosulfate, and sulfide. First, we monitored the growth of strain CS1 when cultured in an SRM medium containing these sulfur sources, compared to the control SRM medium without additional sulfur sources. The dynamics of growth were monitored for 2 months. Moreover, strain CS1 showed different responses to different sulfur sources. Supplementation of 40 mM Na_2_S_2_O_3_, 10 mM Na_2_SO_3_, or 10 mM Na_2_S generally inhibited bacterial growth at the beginning of the incubation period (Figure 2A). After 14 days, the absorbance value (OD_600_) of strain CS1 in 40 mM Na_2_S_2_O_3_ or 10 mM Na_2_S treatment reached a level similar to that of the control group (Figure 2A). And the OD_600_ of strain CS1 in the 40 mM Na_2_S_2_O_3_ group would continue to increase after 21 days and peak at twofold of the control group. Compared to the control group, we also found that adding additional 20 mM sulfate did not significantly increase the growth of strain CS1 (Figure 2A). In addition, cells of strain CS1 incubated with Na_2_SO_3_ or Na_2_S became more elongated compared to control groups or Na_2_SO_4_ and Na_2_S_2_O_3_ treatments (Figure 2).

To understand the mechanisms underpinning the response of CS1 to different sulfur sources, we conducted a proteomic analysis of strain CS1 cultured in a medium supplemented with Na_2_S_2_O_3_, Na_2_SO_3_, or Na_2_S, respectively. Since the growth of *O*. *marinus* CS1 varied in different sulfur sources, we collected bacterial cells for proteomic analysis when the OD_600_ value was about 0.08–0.1 for all samples. Proteomic analysis showed that compared to the control group, a total of 1070, 1255, and 1012 proteins were significantly differentially expressed in the experimental groups containing Na_2_S_2_O_3_, Na_2_SO_3_, and Na_2_S (*p* < 0.05), respectively. Notably, most key enzymes associated with dissimilatory sulfate reduction (such as QmoA, QmoB, QmoC, AprA, AprB, Sat, DsrA, and DsrB) were downregulated when strain CS1 was cultured in a medium supplemented with Na_2_S_2_O_3_, Na_2_SO_3_, and Na_2_S (Figure 2G), indicating that supplementation inhibited the process of dissimilatory sulfate reduction in strain CS1. In contrast, the expression of proteins related to thiosulfate and sulfide metabolism, such as PhsA, MetB (cystathionine gamma‐synthase family protein), and SQR, was significantly upregulated in at least two of the conditions when strain CS1 was cultured in medium supplemented with Na_2_S_2_O_3_, Na_2_SO_3_, or Na_2_S (Figure 2G).

### *O. marinus* CS1 produces ZVS via metabolizing thiosulfate

**Figure 3:**
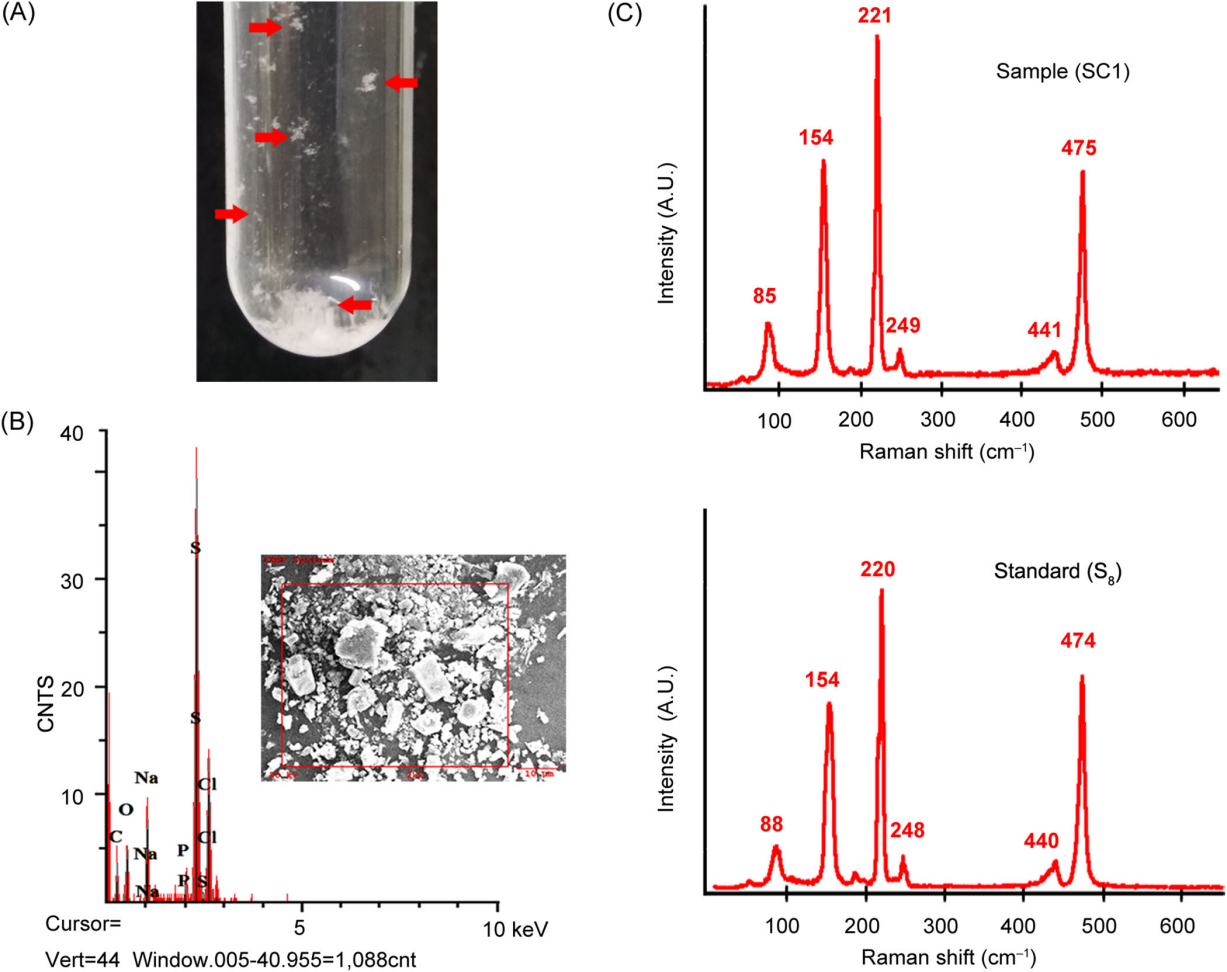
*Oceanidesulfovibrio marinus* CS1 produces ZVS when cultured in a medium supplemented with 40 mM Na_2_S_2_O_3_. (A) Formation of an obvious white substance by *O*. *marinus* CS1 when cultured in a medium supplemented with 40 mM Na_2_S_2_O_3_ (indicated with red arrows). (B) Identification of the major sulfur composition of the white substance produced by *O*. *marinus* CS1 via energy‐dispersive spectrum analysis. The inset shows SEM micrograph of the white substance produced by *O*. *marinus* CS1. (C) Confirmation of S_8_ (standard) configuration of white substance produced by *O*. *marinus* CS1 (Sample CS1) via Raman spectroscopy. ZVS, zero‐valent sulfur.

**Figure 4:**
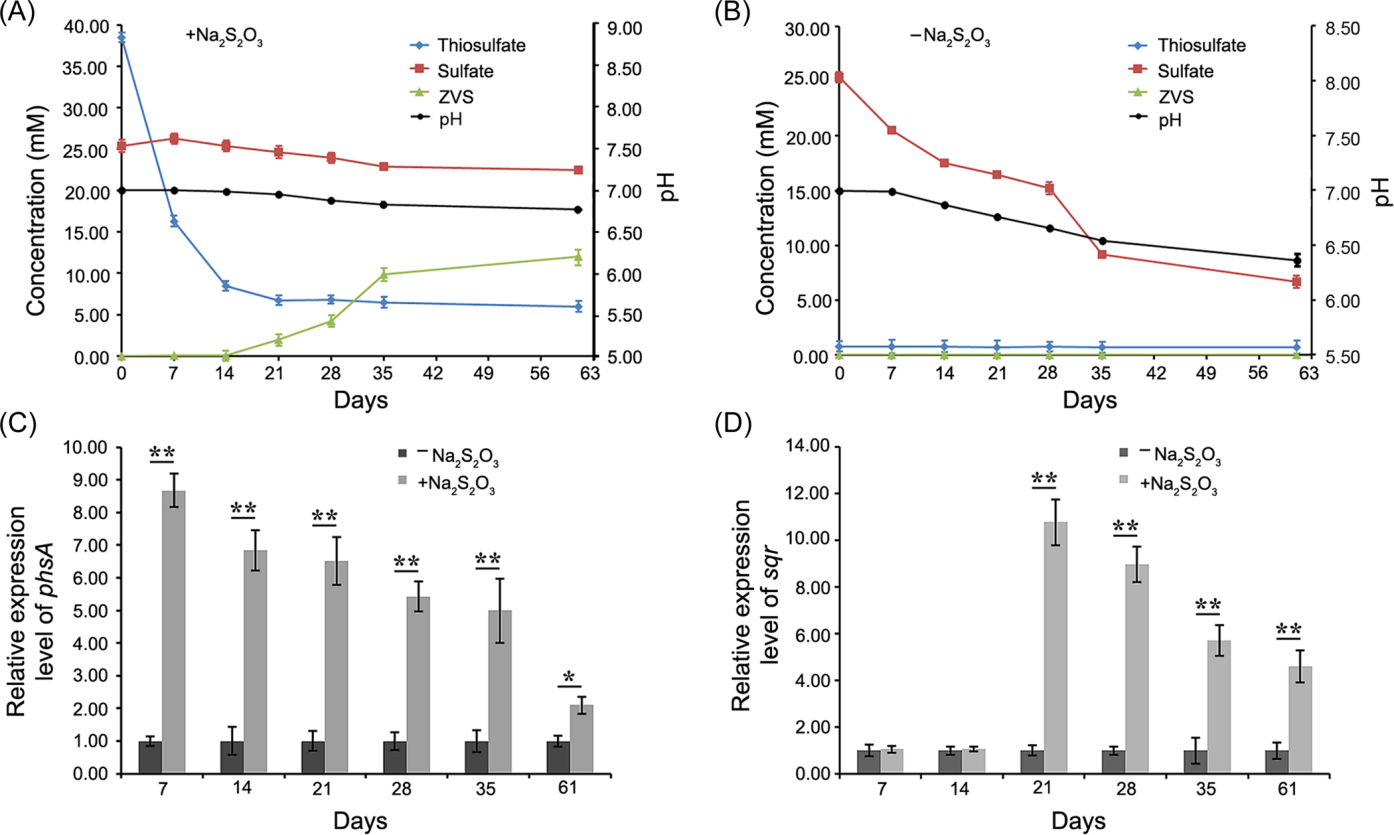
Monitoring the dynamics of different sulfur intermediates and the expression levels of *phsA* and *sqr* in a medium supplemented with 40 mM Na_2_S_2_O_3_. Dynamics of concentrations of sulfate, thiosulfate, ZVS, and pH in medium supplemented with (A) or without (B) 40 mM Na_2_S_2_O_3_ across a 2‐month incubation period. The error bars indicate the standard deviation from three biological replicates. Dynamics of the relative expression levels of *phsA* (C) and *sqr* (D) by qRT‐PCR in a medium supplemented with or without 40 mM Na_2_S_2_O_3_ across the whole 2‐month incubation period is shown. All data are relative to the expression levels found in the control group ± the standard error (*N* = 4). *p < 0.05, **p < 0.01.

An obvious white precipitate was observed when *O. marinus* CS1 was cultured for about 20 days in a medium supplemented with 40 mM Na_2_S_2_O_3_ (Figure 3A). In a previous study, we isolated a deep‐sea bacterium called *Erythrobacter flavus* 21‐3 from the same site as strain CS1, which could oxidize Na_2_S_2_O_3_ to form ZVS through a novel sulfur oxidation pathway^18^. To clarify whether the white substance produced by strain CS1 was also ZVS, SEM and energy‐dispersive spectrum (EDS) assays were initially conducted. SEM showed that the white substance formed regular crystals, which were further identified as elemental sulfur by EDS (Figure 3B). Additionally, Raman spectroscopy of the white substance produced by *O. marinus* CS1 identified three strong peaks at 154, 221, and 475 cm^−1^ (Figure 3C). According to the cyclooctasulfur standard, these peaks correspond to the bending and stretching modes of the eightfold ring, and are typical characteristics of S_8_ (Figure 3C)^7^, ^9^, ^18^. Meanwhile, the formation of ZVS mediated by strain CS1 was tracked across a whole 2‐month incubation period in a medium supplemented with thiosulfate. This showed that ZVS could be detected after 3 weeks of incubation and still increased after 5 weeks of incubation (Figure 4A); a similar pattern to the growth curve of strain CS1 when grown in a medium supplemented with thiosulfate (Figure 2A). Accordingly, the concentration of thiosulfate decreased along with the formation of ZVS, while the concentration of sulfate almost remained unchanged (Figure 4A). In comparison, strain CS1 could not form any ZVS in the medium without extra thiosulfate, while the concentration of sulfate decreased along with bacterial growth (Figure 4B). The above results indicate that strain CS1 drives the formation of ZVS via metabolizing thiosulfate.

### PhsA and SQR play key roles in driving ZVS formation in *O. marinus* CS1

Next, we wanted to find what determines the formation of ZVS in strain CS1 in the presence of thiosulfate. Given that PhsA and SQR were almost expressed in strain CS1 and upregulated upon supplementation with different sulfur sources (Figure 2G), we speculated that PhsA might metabolize thiosulfate to sulfide, which, in turn, is oxidized to ZVS by SQR. To test this hypothesis, we analyzed the dynamics of the expression levels of *phsA* and *sqr*, along with the formation of ZVS as shown in Figure 4A. This showed that the expression level of *phsA* decreased throughout the incubation period, although it was also significantly upregulated in the group added with 40 mM thiosulfate when compared to the control group (Figure 4C). On the other hand, the expression level of *sqr* was only markedly upregulated after 3 weeks of incubation in the 40 mM thiosulfate group when compared to the control group, and from then decreased until the end of the incubation period (Figure 4D)—a similar result to ZVS formation as shown in Figure 4A. The above results are consistent with our speculation that PhsA and SQR catalyze thiosulfate to form sulfide and then ZVS.

**Figure 5:**
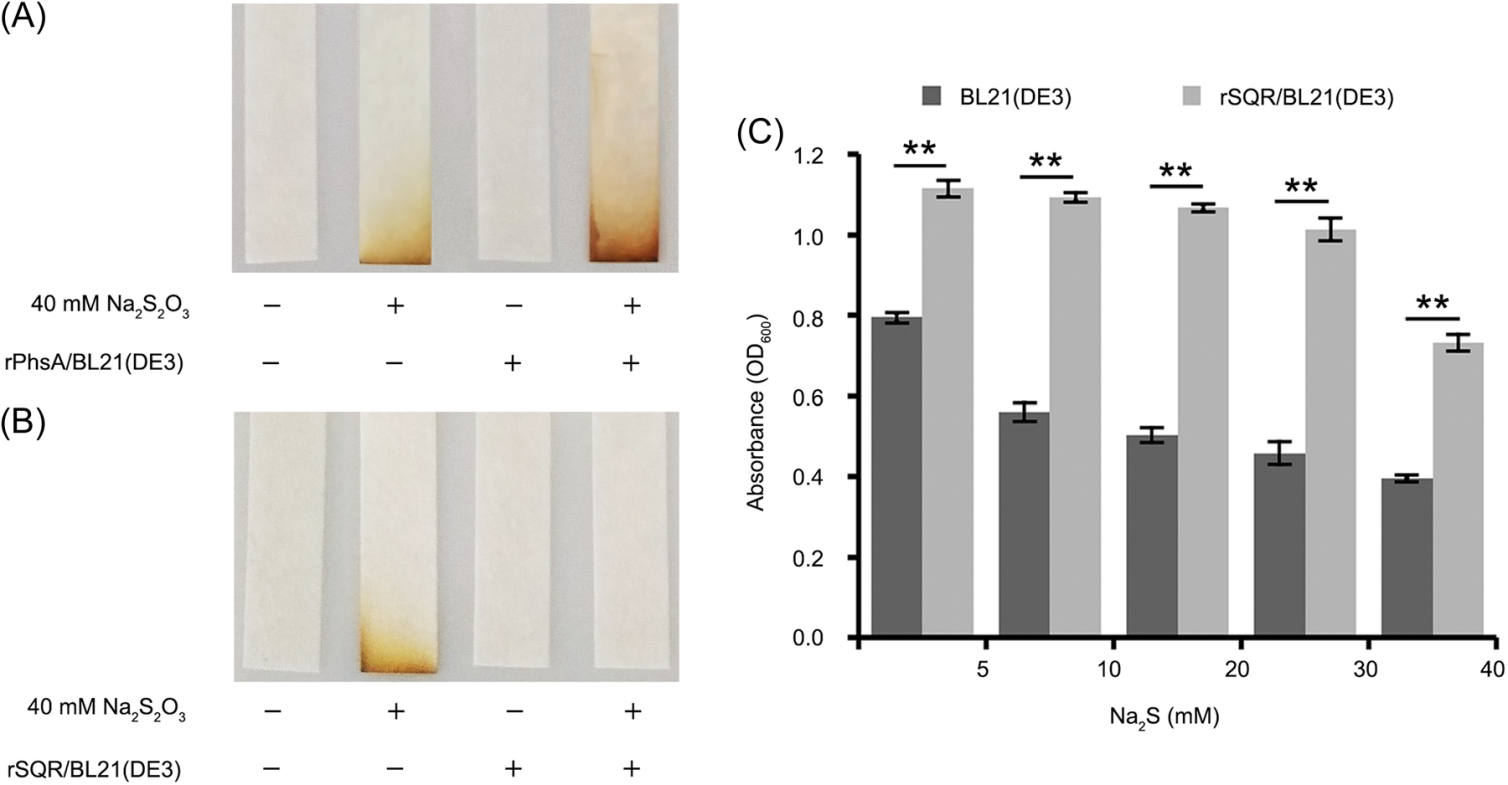
Functional assays of key proteins driving the formation of ZVS in *Oceanidesulfovibrio marinus* CS1. (A) Overexpression of PhsA in *Escherichia coli* promotes the transformation of S_2_O_3_^2−^ to H_2_S. *E*. *coli* cells without or with an expression of PhsA were incubated in an LB medium supplemented with 40 mM Na_2_S_2_O_3_ for 24 h. H_2_S accumulation was detected with lead‐acetate paper strips. (B) Overexpression of SQR in *E*. *coli* promotes the transformation of H_2_S to other forms. *E*. *coli* cells without or with an expression of SQR were incubated in an LB medium supplemented with 40 mM Na_2_S_2_O_3_ for 24 h. H_2_S accumulation was detected with lead‐acetate paper strips. (C) Growth of *E*. *coli* cells either with or without expression of SQR in LB medium supplemented with 5, 10, 20, 30, or 40 mM Na_2_S for 24 h. LB, Luria Bertani; SQR, quinone oxidoreductase. **p < 0.01.

Given the absence of a genetic operation system of strain CS1, we further verified the above functions of PhsA and SQR in *Escherichia coli*. First, we overexpressed *O*. *marinus* CS1 *phsA* (E8L03_06385) and *sqr* (E8L03_05425) in *E*. *coli* BL21(DE3) (Figure S2). PhsA has been shown to catalyze the decomposition of thiosulfate into sulfite and H_2_S^43^, ^44^, ^45^. Accordingly, overexpression of PhsA in *E. coli* significantly promoted the production of H_2_S from added thiosulfate (Figure 5A), indicating that PhsA of strain CS1 functions as an enzyme that catalyzes thiosulfate reduction to H_2_S. Notably, a similar *phs* operon has been confirmed to exist in *E. coli* ^46^, correspondingly, a background level of H_2_S is also detected in wild‐type *E*. *coli* BL21(DE3) supplemented with 40 mM Na_2_S_2_O_3_ (Figure 5A,B). *E*. *coli* BL21(DE3) overexpressing PhsA could obviously produce more H_2_S than that in the wild type (Figure 5A).

SQR has the potential to oxidize sulfide to ZVS, as shown in Figure 1D. As expected, the overexpression of SQR could efficiently remove H_2_S produced by *E. coli* when cultured in a medium supplemented with 40 mM Na_2_S_2_O_3_ (Figure 5B), benefiting the bacterial cells by alleviating the toxic effects of H_2_S. We thus further analyzed the activity of sulfide oxidation mediated by SQR in *E*. *coli* BL21(DE3) cultured in a medium supplemented with different concentrations of Na_2_S (5, 10, 20, 30, and 40 mM). If SQR could convert sulfide to ZVS, the toxicity of sulfide to *E. coli* cells would be significantly reduced. Indeed, overexpression of SQR in *E*. *coli* BL21(DE3) significantly promoted (*p* < 0.01) bacterial growth when compared with the control group, regardless of the concentration of Na_2_S in the medium (Figure 5C). Therefore, we propose that PhsA and SQR might drive ZVS formation in strain CS1 by catalyzing thiosulfate reduction and sulfide oxidation.

As SQR is a key enzyme catalyzing sulfide oxidation to form ZVS, we further analyzed SQR homologs identified in different microbes. In total, six types (Type I–VI) have been identified in bacteria, archaea, and eukaryotes^20^. Based on phylogenetic analysis, SQR in *O*. *marinus* CS1 was clustered into the branch of Type III SQRs with two conserved amino acid residues at Cys159 and Cys331 (Figure S3). Homologous sequences of SQR in strain CS1 were also identified in other species belonging to sulfate‐reducing *Deltaproteobacteria*, including *Oceanidesulfovibrio indonesiensis*, *Desulfohalovibrio alkalitolerans*, *Desulfatirhabdium butyrativorans*, *Desulfospira joergensenii*, *Pseudodesulfovibrio* sp. SRB007, and *Pseudodesulfovibrio* sp. zrk46 (Figure S3). It is notable that strains SRB007 and zrk46 are two deep‐sea SRB that were isolated from the same sampling site as strain CS1.

### Proteomic analysis of sulfur metabolism by *O*. *marinus* CS1 in the deep sea

**Figure 6:**
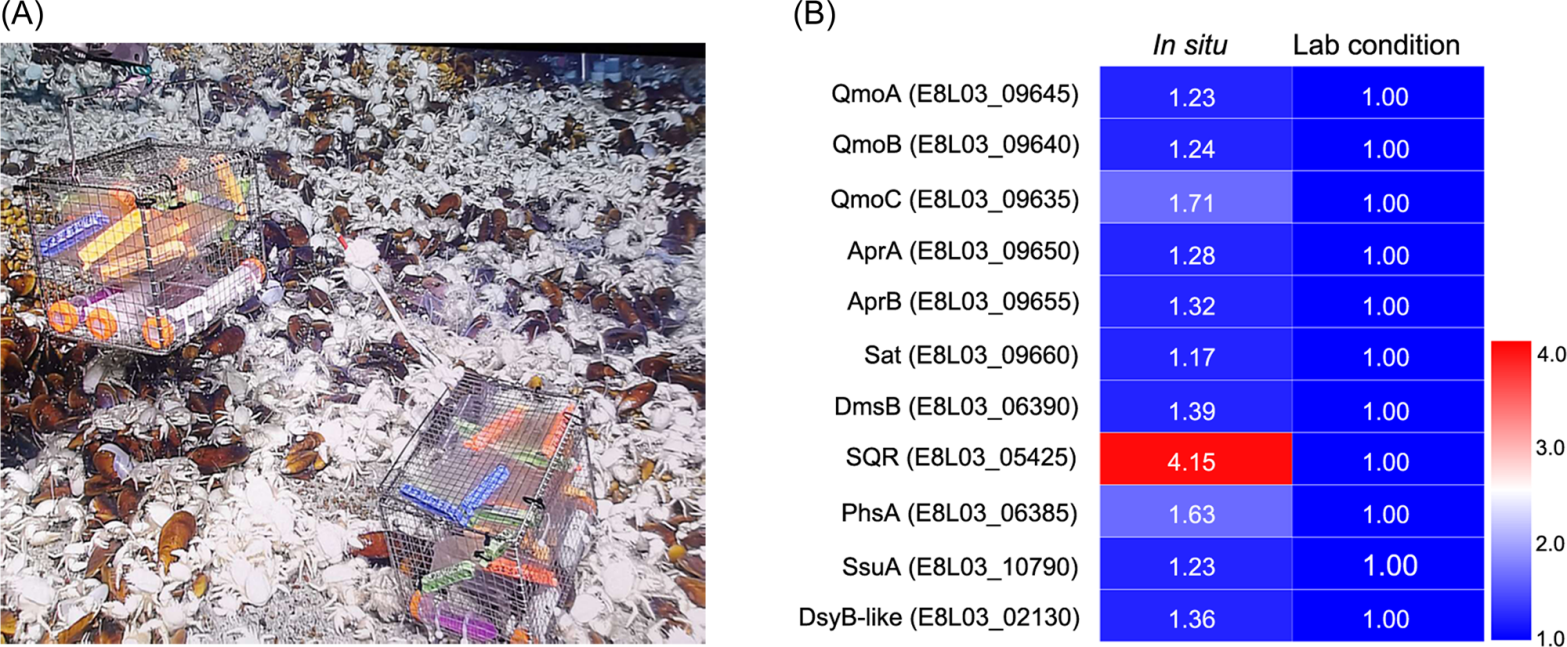
Proteomic analysis of sulfur metabolism of *Oceanidesulfovibrio marinus* CS1 cultured in a deep‐sea cold seep. (A) Representative picture of the in situ experimental apparatus used in the deep‐sea cold seep. (B) Heatmap of differentially expressed proteins involved in sulfur metabolism after a 10‐day incubation of *O*. *marinus* CS1 in the in situ group compared with laboratory conditions. The numbers in the heatmap represent the fold change of protein expression compared to the laboratory conditions group. Other abbreviations are the same as in Figures 1 and 2. More detailed information about the protein expression shown in this figure is listed in Supporting Information: Dataset Sheets S2 and S4. DsyB, MTHB methyltransferase/dimethylsulfoniopropionate biosynthesis enzyme.

As shown above, *O*. *marinus* CS1 responded to different sulfur‐containing compounds and formed ZVS in laboratory conditions. Given that strain CS1 is a typical deep‐sea SRB, it is necessary to explore its sulfur metabolism in an environment like the deep sea. Taking advantage of a cruise in May 2020, we therefore placed the strain CS1 in deep‐sea cold seep for 10 days in dialysis bags (Figure 6A). This site had similar environmental parameters to the site at which strain CS1 was isolated (Table S1). After 10 days of incubation, bacterial cells in the “in situ” group and control group (incubated in the laboratory conditions) were collected and proteomic analyses were performed after verification of their purity. As expected, proteomic data showed that the expression of most key proteins associated with sulfate reduction (both assimilatory and dissimilatory) was significantly upregulated in the “in situ” group compared to those in laboratory conditions (Figure 6B), strongly indicating that sulfate reduction is required for strain CS1 to thrive in the deep‐sea. Surprisingly, the expression of SQR was the most upregulated in the “in situ” group compared to laboratory conditions (Figure 6B). Given that SQR was also significantly upregulated when stimulating strain CS1 with thiosulfate (Figure 4D) and sulfite (Figure 2G middle panel) in the laboratory, and that SQR is broadly distributed in different bacteria (Figure S3), we propose that SQR might play an essential role in driving sulfide oxidation in strain CS1 and other microbes. Expression of PhsA was also evidently upregulated in the “in situ” group (Figure 6B), indicating thiosulfate metabolization may be a major metabolic pathway for strain CS1 in the deep‐sea environment. Given the fact that the expressions of SQR and PhsA were simultaneously upregulated, we speculate that strain CS1 might form ZVS in the deep‐sea environment.

**Figure 7:**
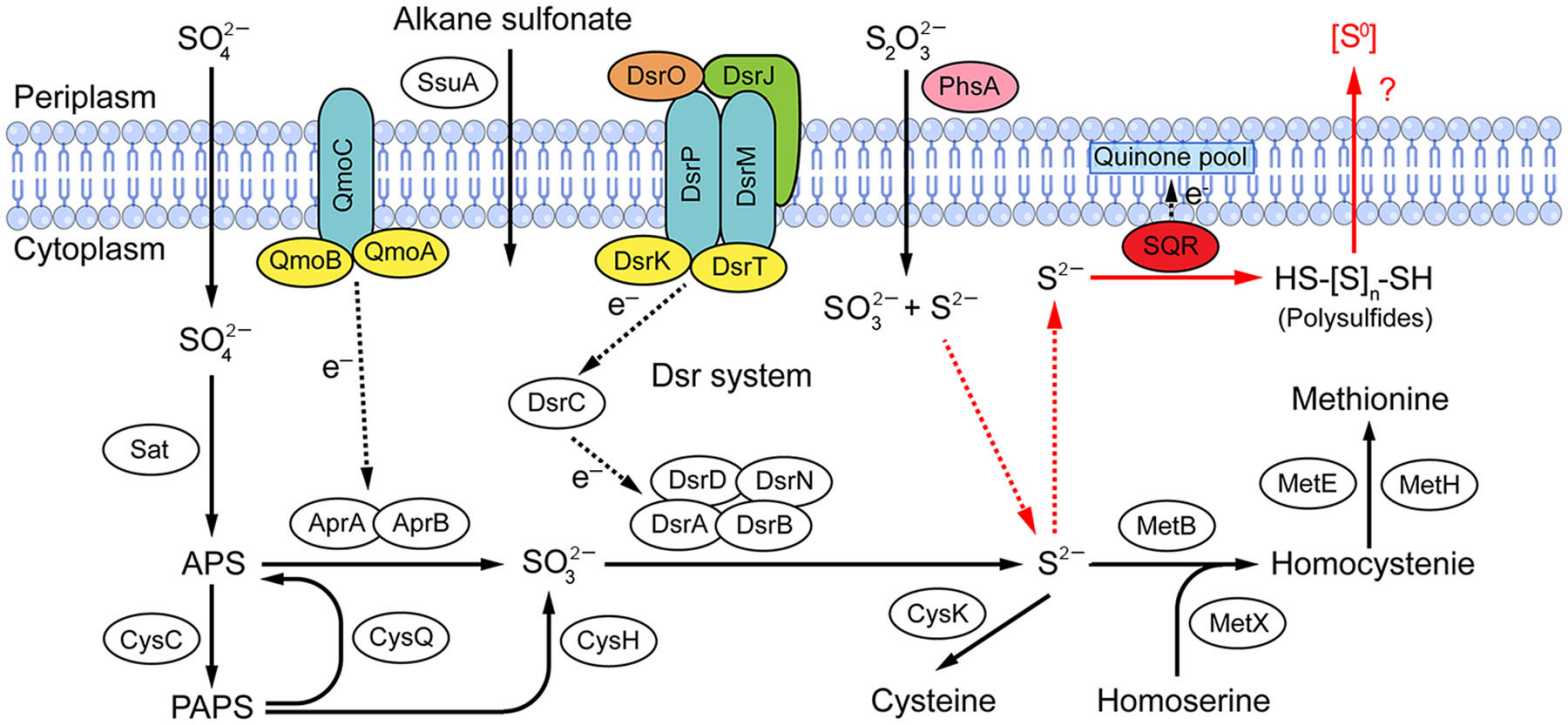
Proposed model of sulfur metabolism and ZVS formation in *Oceanidesulfovibrio marinus* CS1. Black solid lines represent the typical sulfate reduction pathway present in *O*. *marinus* CS1. Black dashed lines represent the direction of electron transfer. Red lines represent the unique ZVS formation process present in *O*. *marinus* CS1. Other abbreviations are the same as shown in Figures 1, 2, and 6. More detailed information about the proteins shown in this figure is listed in Supporting Information: Dataset Sheet S2. MetE, 5ʹ‐methyltetrahydropteroyltriglutamate‐homocysteine *S*‐methyltransferase; MetH, methylenetetrahydrofolate reductase [NAD(P)H].

Overall, based on our present results, we suggest a model for central sulfur metabolism in *O*. *marinus* CS1 (Figure 7). First, sulfate is transported into the cells and then reduced to sulfite through both dissimilatory and assimilatory reduction pathways. Thereafter, sulfite is further reduced to sulfide mediated by the DSR complex via a typical sulfite dissimilatory reduction pathway. Meanwhile, thiosulfate is reduced to sulfide by PhsA. Finally, a small part of the generated sulfide is used for sulfur‐containing amino acid (e.g., cysteine and methionine) synthesis^47^, and others could be oxidized to polysulfide or even ZVS by SQR. ZVS is finally exported outside of cells.

### SRB potentially contribute to the formation of ZVS in deep sea

**Figure 8:**
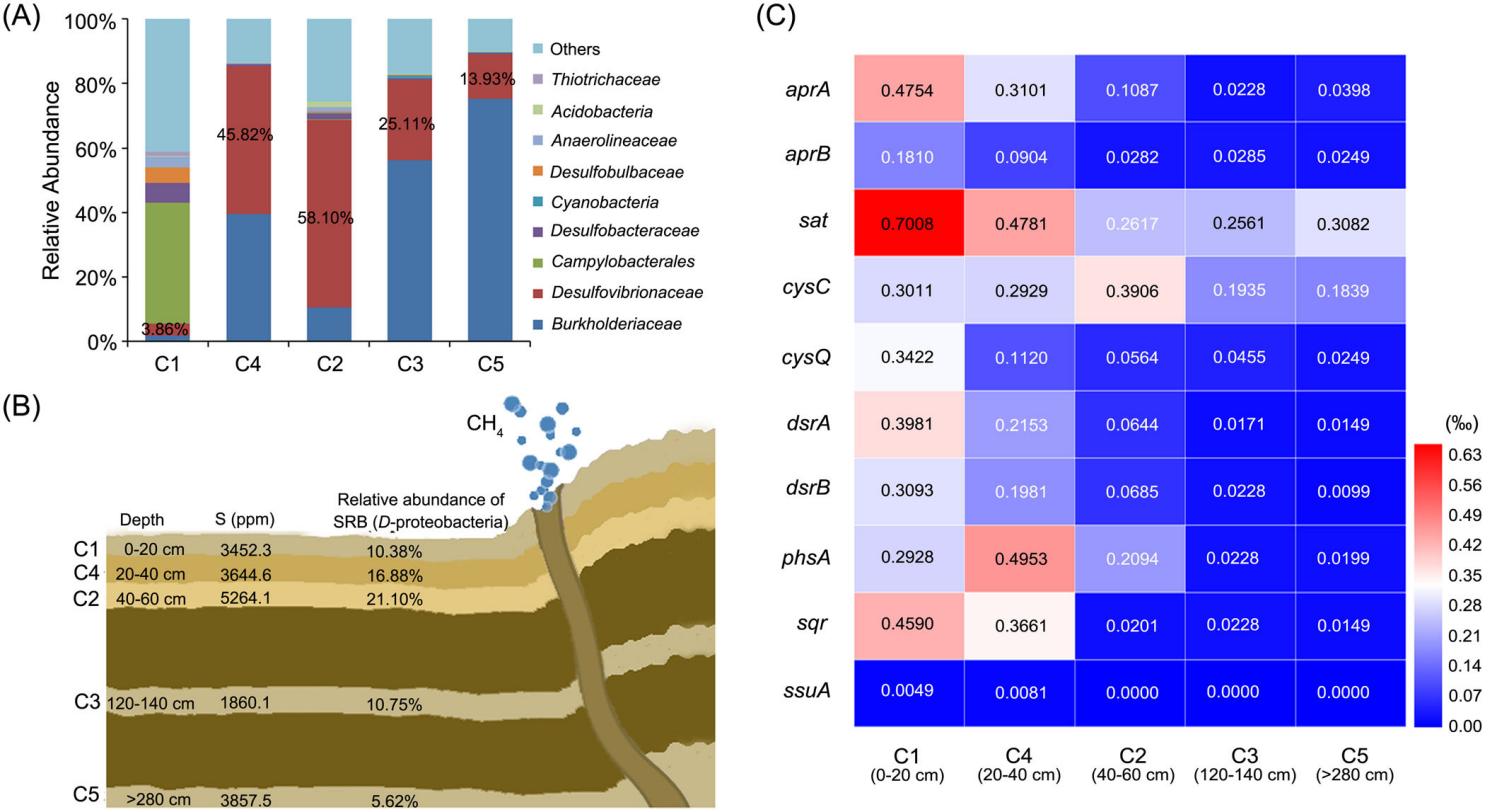
Metagenomic analyses of the abundance of deep‐sea SRB and corresponding key genes involved in the sulfur cycle. (A) The relative abundance of *Desulfovibrionaceae* bacteria in the different depths of cold seep sediments is based on 16S rRNA amplicon data. (B) The distribution of sulfate‐reducing *Deltaproteobacteria* in different depths of cold seep sediments based on metagenomic data. S (ppm) represents the concentration of sulfur in different depths of sediments based on X‐ray fluorescence spectrometry. The percentage represents the relative proportion of functional genes derived from sulfate‐reducing Deltaproteobacteria. (C) Key genes involved in the sulfur cycle, as well as ZVS formation, are identified in metagenomic data derived from the deep‐sea cold seep sediments.

As shown above, *O*. *marinus* CS1 forms ZVS in laboratory conditions and possibly in the deep‐sea environment. We next intended to clarify the relative abundance of sulfate‐reducing *Deltaproteobacteria* in deep‐sea cold seeps and their potential to form ZVS. The relative abundance of sulfate‐reducing *Desulfovibrionaceae* was thus investigated by using the operational taxonomic units (OTUs) method with samples collected from different depths of sediments. Bacteria from *Deltaproteobacteria* family were detected to have high relative abundance in each sample of cold seep sediments (Figure 8A). According to the results, the relative abundances of the family *Desulfovibrionaceae* in the whole bacterial community in deeper sediments (C4, C2, C3, C5) were significantly higher than that in surface sediment (C1). Among them, family *Desulfovibrionaceae* bacteria in the C4 (20–40 cm) and C2 (40–60 cm) samples accounted for higher relative abundances of 45.82% and 58.10%, respectively, indicating that the cold seep sediments at a depth of 20–60 cm were the main habitats of the family *Desulfovibrionaceae* (Figure 8A). To obtain deeper insights into the distribution of genes associated with ZVS production in sulfate‐reducing *Deltaproteobacteria*, metagenomic sequencing was performed with samples collected at different depths from the sedimental surface. As expected, sequences associated with sulfate‐reducing *Deltaproteobacteria* by taxonomic annotation had a higher relative abundance in the cold seep sediments, accounting for 10.38%, 16.88%, 21.10%, 10.75%, and 5.62% of the whole bacterial community in the samples C1, C4, C2, C3, and C5, respectively (Figure 8B). After careful annotation and analyses of genes obtained from the metagenomic sequencing, we found that key genes responsible for both sulfate dissimilatory and assimilatory reduction pathways were distributed in the metagenomes of sulfate‐reducing *Deltaproteobacteria* and other bacteria in different samples (Figure 8C and Supporting Information: Dataset Sheet S6). *sqr* and *phsA* could be identified in different samples in relatively high proportions, strongly indicating that the sulfate‐reducing *Deltaproteobacteria* in different depths of sediment have the potential to form ZVS.

## DISCUSSION

ZVS, in the form of elemental sulfur and dissolved polysulfide sulfur, is commonly found in the highly reducing, sulfidic environments that characterize AOM ecosystems including cold seeps^29^. SRB are an important population inhabiting cold seeps and have a crucial role in the sulfur cycle, from which the formation of ZVS represents a novel pathway^28^. ZVS was proposed to be generated by SRB under unfavorable conditions, for example, high concentrations of sulfide that are inhibitory^32^. Hence, sulfide is a key intermediate for SRB to produce ZVS. It is notable that sulfide could be generated from thiosulfate via a reduction process catalyzed by PhsA, a kind of thiosulfate reductase (Figure 1D). Therefore, it is possible that SRB can also generate ZVS from thiosulfate. However, until now no studies have shown ZVS production from thiosulfate mediated by SRB. In the present study, we report for the first time that *O*. *marinus* CS1, a typical deep‐sea SRB, generates ZVS from thiosulfate, mediated by PhsA and SQR. In this process, PhsA catalyzes thiosulfate to form sulfide, which is then oxidized by SQR to form ZVS.

Thiosulfate has been mentioned as an important shunt in marine environments for the coupling of reductive, oxidative, and disproportionation pathways of the sulfur cycle^36^. The reduction of thiosulfate is one of the important processes for anaerobic energy metabolism of SRB in deep‐sea marine sediments^26^, ^48^. In surface marine sediments (0–10 cm in depth), a total of 15%–50% of thiosulfate is reduced by sulfate‐reducing microorganisms, and approximately 30%–60% of sulfide is produced during this process^36^. PhsA and its homologs are crucial for catalyzing thiosulfate to sulfide, which contributes greatly to an intraspecies sulfur cycle that drives S_0_ respiration in different bacteria^43^, ^46^. We identified *phsA* in the genome of *O*. *marinus* CS1 and proposed it encodes PhsA protein to reduce thiosulfate to sulfide (Figure 1D). Indeed, its expression level was significantly upregulated in medium supplemented with different sulfur sources in the laboratory (Figure 2G) and deep‐sea in situ conditions (Figure 6C,D), strongly indicating it is essential for sulfur cycling by *O*. *marinus* CS1. Its expression was upregulated across the whole 2‐month incubation period in the presence of thiosulfate (Figure 4C) and it is indispensable for strain CS1 to reduce thiosulfate to sulfide. Overexpression of PhsA in *E. coli* also promoted the conversion of thiosulfate to sulfide (Figure 5A).

It is known that sulfide is a highly toxic compound for microorganisms and eukaryotes^49^, ^50^. On the other hand, sulfide is also an important intermediate in the geochemical cycle of the sulfur element, and microbes have evolved different strategies to transform it into other forms to relieve the potential toxicity. Indeed, the addition of 10 mM Na_2_S significantly slowed down the growth of strain CS1, and it took a long time for the bacterial cells to remove the toxic effects of sulfide (Figure 2). In addition, significant changes in the morphology of strain CS1 were observed after supplemented with sulfide or sulfite. Since cellular morphology is an important characteristic in the study of bacterial physiology, which has a complicated relationship with the growth rate and metabolism of cells^51^, ^52^. Although we still do not know the specific mechanism by which the addition of sulfide and sulfite changes the morphology of strain CS1 cells, it is certain that the cellular morphology of *O*. *marinus* CS1 must be closely related to the metabolic processes of different sulfur compounds.

The key protein in the metabolic pathway is usually an important molecule to clarify this process of bacteria. SQR, a sulfide oxidoreductase, enables cells to oxidize sulfide to ZVS and can potentially alleviate the toxicity of sulfide^20^. Accordingly, we identified a gene encoding SQR in the genome of strain CS1 (Figure 1D). Notably, the expression dynamics of SQR showed a very similar pattern to the formation of ZVS when strain CS1 was cultured in a medium supplemented with thiosulfate (Figure 4A,D), indicating that SQR is closely related to the formation of ZVS from thiosulfate in strain CS1. Unfortunately, the significant differential expression of SQR protein was not detected in the experimental group containing thiosulfate according to the proteomics results (Figure 2G). The main reason should be due to the difference in sample collection time. In the group containing thiosulfate, cells of strain CS1 were collected on the 14th day with an OD_600_ value of 0.08–0.1. However, the transcription level of the *sqr* gene had not been significantly upregulated at this time according to the result of quantitative polymerase chain reaction (qPCR) (Figure 4D). Therefore, we speculate that SQR in strain CS1 oxidizes sulfide to ZVS for detoxification. This was confirmed by the effects of SQR when overexpressed in *E*. *coli* BL21(DE3) cells, in which it reduced the toxicity of Na_2_S in different concentrations, ranging from 5 to 40 mM (Figure 5). It is notable that another member of the *Desulfovibrionaceae* family (*Desulfovibrio pigers* Vib‐7) could not grow in a medium containing 6 mM or higher sulfide^53^, while strain CS1 could tolerate up to 10 mM of sulfide (Figure 2A). Interestingly, the genome of *D*. *piger* (LT630450.1) does not contain an SQR homolog.

Although thiosulfate was reduced to sulfite and sulfide by PhsA, the production of ZVS was not found when the above two sulfur sources were added separately in the culture medium of *O*. *marinus* strain CS1 (Figure 2A). This shows that strain CS1 may only produce ZVS when the thiosulfate exists. Therefore, the thiosulfate is like a decisive molecule, and its presence initiates the reaction process for the production of ZVS in strain CS1. In addition, adding high concentrations (40 mM) of thiosulfate had been found to inhibit the dissimilatory sulfate reduction pathway of strain CS1 (Figure 2G). This result is contrary to the newly proposed hypothesis that SRB generates ZVS through the dissimilatory sulfate reduction^28^, ^32^. In summary, this may be another novel pathway to produce ZVS in SRB.

Notably, in the deep‐sea in situ environment, the expression of both PhsA and SQR was markedly upregulated (Figure 6B). Due to time limitations, we were unable to culture *O*. *marinus* CS1 for a longer time (e.g., up to 60 days) to observe the formation of ZVS. Through metagenomic analysis, we found homologs of strain CS1 SQR broadly distributed in other SRB species inhabiting the same cold seep environment of the South China Sea (Figure S4 and Supporting Information: Dataset Sheet S3). Moreover, homologs of key proteins (including PhsA and SQR) involved in the sulfur cycle in *O*. *marinus* CS1 were found widely in the metagenomes of SRB and other bacteria that live in the sediments of the South China Sea (Figure 8C). Therefore, we propose that a novel ZVS formation pathway for thiosulfate metabolism, mediated by PhsA and SQR, is used by many SRB or even other bacteria in deep‐sea environments.

## MATERIALS AND METHODS

### Isolation and cultivation of *O. marinus* CS1

To isolate SRB from the deep‐sea cold seep, sediment samples were collected by R/V *KEXUE* in the South China Sea (119°17ʹ04.956ʹʹ E, 22°06ʹ58.384ʹʹ N) at a depth of approximately 1143 m in September 2017 (Table S1). The microorganisms in these samples were cultured by using the modified SRM that contained 6.5 g PIPES (C_8_H_18_N_2_O_6_S_2_), 4.3 g MgCl_2_·6H_2_O, 0.25 g NH_4_Cl, 0.5 g KCl, 0.14 g CaCl_2_, 0.14 g K_2_HPO_4_·3H_2_O, 0.001 g Fe(NH_4_)_2_(SO_4_)_2_·6H_2_O, 0.1 g CH_3_COONa (acetate), 2.24 g CH_3_CHOHCOONa (lactate), 20 mM absolute ethanol, 1 ml trace element solution (Table S2), 0.5 g cysteine, and 0.001 g resazurin in 1 l filtered seawater, and 15 g/l agar was added to prepare the corresponding solid medium, pH 7.0. Then the medium was sparged with 80% N_2_ and 20% CO_2_ gas mixture for 30 min to make it anoxic. After a month of anaerobic enrichment at standard atmospheric pressure, a 50 µl culture was spread on the solid SRM medium prepared in the Hungate tubes, which were further cultured at 28°C for 7 days in the anaerobic incubator (LAI‐3; Longyue). Individual colonies were picked respectively by sterilized bamboo sticks and incubated in the SRM broth. Strain CS1 was purified by the Hungate roll‐tube method until considered to be a pure culture. Genomic DNA extraction and PCR amplification of the 16S rRNA gene sequence of strain CS1 were performed as previously described^18^.

### Electron microscopic analysis

The morphological characteristics of *O*. *marinus* CS1 were observed by SEM (S‐3400N; Hitachi) and TEM (HT7700; Hitachi). The ZVS produced by strain CS1 in medium supplemented with Na_2_S_2_O_3_ was identified via EDS (model 550i; IXRF systems) equipment with SEM and Raman spectra confocal microscope (WITec alpha300 R system; WITec Company), respectively, as described in our previous study^18^. After incubation in a medium supplemented with 40 mM Na_2_S_2_O_3_ for 30 days, the milky white supernatant in strain CS1 medium was collected by centrifugation (5000*g*, 10 min) and lyophilized, then the pellet was used for EDS analysis at an accelerating voltage of 5 keV for 30 s and Raman spectra.

### Genome sequencing and annotation

Genomic DNA was extracted from *O*. *marinus* CS1 that was cultured for 7 days at 28°C. The harvested DNA was detected by the agarose gel electrophoresis and quantified by Qubit 3.0 (Thermo Fisher Scientific). Whole‐genome sequence determinations of strain CS1 were carried out with the PacBio (Pacific Biosciences) and Illumina MiSeq (Illumina) sequencing platforms. The genome of strain CS1 was sequenced by the PacBio platform (PacBio) using single molecule real‐time (SMRT) technology, which was performed at the Beijing Novogene Bioinformatics Technology Co., Ltd. The low‐quality reads were filtered by the SMRT Link v5.0.1 and the filtered reads were assembled to generate one contig without gaps and were manually circularized by deleting an overlapping end.

The genome relatedness values were calculated by ANI based on the BLASTN algorithm with recommended species criterion cut‐offs of 95% (JSpecies WS, http://jspecies.ribohost.com/jspeciesws/) and the AAI based on AAI‐profiler with values above 95%–97% corresponding to the same species (http://ekhidna2.biocenter.helsinki.fi/AAI/). To determine the phylogenetic position of *O*. *marinus* CS1, the 16S rRNA gene sequence was aligned by the BLAST program (https://blast.ncbi.nlm.nih.gov/Blast.cgi), and the phylogenetic tree was constructed with MEGA X^54^.

### Growth assays of *O*. *marinus* CS1 in medium supplemented with different sulfur sources

Growth of strain CS1 was performed under the standard atmospheric pressure. Briefly, 15 ml fresh *O*. *marinus* CS1 was cultured in SRM supplemented with or without 20 mM Na_2_SO_4_, 40 mM Na_2_S_2_O_3_, and 10 mM Na_2_SO_3_ for 2 months at 28°C in 2 l anaerobic bottles (the pH value in each culture condition is adjusted to 7.0), respectively. Furthermore, to analyze whether strain CS1 could directly oxidize sulfide or resist the toxicity of sulfide, 10 mM Na_2_S was added to SRM as described above. Each condition had three replicates. Bacterial growth status was monitored by measuring the absorbance value at 600 nm (OD_600_) via the Infinite M1000 Pro ultraviolet–visible (UV–vis) spectrometer (Tecan). For the morphological observation, cells of strain CS1 with OD_600_ values at 0.08–0.1 were collected and recorded under the TEM as described above.

### Proteomic analysis

Proteome analysis was performed by PTM BIO (PTM Biolabs Inc.). Briefly, cell suspensions of *O*. *marinus* CS1 with an OD_600_ value of 0.08–0.1 were collected from different groups at different time points: 7 days for the control group and Na_2_SO_4_ supplement group, 14 days for the Na_2_S_2_O_3_ supplement group and Na_2_S supplement group, and 42 days for Na_2_SO_3_ supplement group, respectively. Then, proteomic analysis was performed on cells after checking the purity of the culture by 16S rRNA sequencing to confirm. For proteome analyses, the cells of strain CS1 were sequentially washed with 10 mM phosphate buffer solution (pH 7.4), resuspended in lysis buffer (8 M urea, 1% protease inhibitor cocktail), and disrupted by sonication. The remaining debris was removed by centrifugation at 12,000*g* at 4°C for 10 min. Finally, the supernatant was collected and the protein concentration was determined with a BCA protein assay kit (Thermo Fisher Scientific) according to the manufacturer's instructions. Trypsin digestion, tandem mass tag labeling, high performance liquid chromatography fractionation, liquid chromatography‐tandem mass spectrometry analysis, database search, and bioinformatics analysis are described in the Supporting Information in detail. Analysis of the differentially expressed proteins was performed using HemI software^55^.

To perform the proteomic analysis of cells cultured in the deep‐sea cold seep, strain CS1 was firstly cultured in SRM for 7 days under laboratory conditions and then was separated into two parts: one was equally divided into three dialysis bags (8000–14,000 Da cutoff) as the “in situ” group. This method allowed the exchanges of substances smaller than 8000 Da but prevented bacterial cells from entering or leaving the bag. The other part was equally divided into three anaerobic bottles and incubated for 10 days in the laboratory conditions as the control group. The “in situ” group was simultaneously placed in the same cold seep (E119°17′04.429″, N22°07′01.523″) for 10 days during the cruise of R/V *KEXUE* in May 2020. After 10 days of incubation in the cold seep, the dialysis bags were taken back and the cells were immediately collected and saved at −80°C. A 16S rRNA sequencing was performed to ensure the purity of cells of strain CS1 that were collected from the in situ cultivation. The proteomic assays were performed as described above.

### Analytical techniques for the determination of different sulfur compounds and quantitative real‐time PCR assay

To detect the changes in concentrations of thiosulfate and sulfate, 50 ml cultures of *O*. *marinus* CS1 were collected from groups supplemented with or without 40 mM Na_2_S_2_O_3_ at the 7th, 14th, 21st, 28th, 35th, and 61st day, respectively. Meanwhile, the growth status was monitored. After centrifugation at 12,000*g* at 4°C for 30 min, the concentration of thiosulfate in the supernatant was titrated by a modified iodometry with 0.05 mM K_2_Cr_2_O_7_ standard solution according to previous methods^56^, ^57^, ^58^. Furthermore, the concentration of sulfate in the supernatant was measured by a modified barium sulfate turbidimetry at 420 nm (UV–vis spectrometer, Infinite M1000 Pro; Tecan), which was followed by previous studies^59^, ^60^. The concentration of ZVS (S_8_) in the medium was detected according to the method described previously^61^. Briefly, 1 ml cultured medium was extracted three times using a total of 5 ml dichloromethane. The extracted sample was measured on a UV–vis spectrometer (Infinite M1000 Pro; Tecan) at 270 nm^61^. The detection methods of thiosulfate, sulfate, and ZVS are described in the Supporting Information in detail.

To perform the quantitative real‐time PCR (qRT‐PCR) assay, total RNAs of cells collected at different time points in groups with or without 40 mM Na_2_S_2_O_3_ were extracted with TRIzol reagent (Invitrogen). First‐strand complementary DNA (cDNA) synthesis was carried out with ReverTra Ace® qPCR RT Master Mix (TOYOBO) based on the manufacturer's instructions. The expression levels of *sqr* and *phsA* were determined using qRT‐PCR on different cDNA samples obtained from cultures as described above. Specific primers were designed on the basis of the corresponding sequences in the genome of *O*. *marinus* CS1 CS1 (Table S3). The comparative threshold cycle ($2^{-∆∆C_{t}}$) method was used to analyze the expression level^62^. A pair of 16S rRNA gene primers for *O*. *marinus* CS1 (Table S3) was used as internal controls to check the successful transcription and to calibrate the cDNA template of corresponding samples. qRT‐PCR was performed using a Quant Studio^TM^ 6 Flex (Life Technologies), and the results were analyzed via the system's accompanying SDS software. Dissociation curve analysis was performed to confirm that only one PCR product was amplified and detected. All data were given in terms of relative mRNA expressed as means ± standard error (*N* = 4).

### Functional assays of PhsA and SQR of *O*. *marinus* CS1

To detect the functions of SQR and PhsA that were identified in *O*. *marinus* CS1, the genes encoding these two proteins were respectively cloned and overexpressed in *E*. *coli*. First, the open reading frame of *sqr* or *phsA* was amplified from the *O*. *marinus* CS1 genome using the KOD One^TM^ PCR Master Mix (TOYOBO) with corresponding primers (Table S3). The PCR product was purified by using a DNA Gel Extraction Kit (TsingKe), and then cloned in the plasmid pMD19‐T simple (Takara). The DNA fragment was digested with *Eco*RI/*Xho*I and *Bam*HI/*Eco*RI (Thermo Fisher Scientific), respectively, and ligated into the same restriction enzyme sites of expression vector pET28a (+) (Merck). The recombinant plasmids were transformed into the competent cell *E*. *coli* BL21(DE3) (TsingKe), and transformants were incubated in Luria Bertani (LB) broth (10 g/l NaCl, 10 g/l tryptone, and 5 g/l yeast extract in Milli‐Q water) supplemented with 50 μg/ml kanamycin at 37°C. Protein expression was induced at an OD_600_ around 0.6–0.8 with 0.1 mM isopropyl‐1‐thio‐β‐d‐galactopyranoside (IPTG), and the cells were cultured for further 20 h at 16°C. The resultant proteins were separated by sodium dodecyl‐sulfate polyacrylamide gel electrophoresis and visualized with Coomassie Bright Blue R250 staining.

The lead‐acetate test papers were used to detect the amount of hydrogen sulfide (H_2_S), which reflected the capability of PhsA for reducing thiosulfate to produce H_2_S. *E*. *coli* BL21(DE3) cells overexpressing with recombinant PhsA (rPhsA/BL21(DE3)), SQR (rSQR/BL21(DE3)), and the negative control *E*. *coli* BL21(DE3) were cultured in the medium supplemented with 40 mM Na_2_S_2_O_3_, respectively. After being induced with 0.1 mM IPTG, the production of H_2_S was detected after incubation at 37°C for 24 h. Different concentrations of Na_2_S (5, 10, 20, 30, and 40 mM) was respectively added in LB medium. After being induced with 0.1 mM IPTG, *E*. *coli* BL21(DE3) cells overexpressing with or without rSQR were cultured at 37°C for 24 h. And then the growth statuses of *E*. *coli* rSQR/BL21(DE3) and the negative control *E*. *coli* BL21(DE3) were measured by OD_600_.

### Phylogenetic analysis

To assess the type of SQR in *O*. *marinus* CS1, amino acid sequences of SQR homologs^20^, ^22^ were extracted from NCBI databases (including Type I–VI SQRs) and our metagenomic sequencing data. After sequence alignment, a maximum‐likelihood phylogenetic tree was constructed with the LG+G4+F model (‐bb 1000) using IQ‐TREE. The sequence alignments for all trees were calculated using the MEGA X soft with Clustal W/MUSCLE program^54^. All trees were visualized using iTOL (v5)^63^.

### Metagenomic sequencing and amplicon analysis

For metagenomics analysis, samples were collected from cold seep sediments at a depth of 1146 m in the South China Sea during the cruise of the R/V *KEXUE* in the July of 2018 (Table S4). Samples were collected at depth intervals of 0–20 cm (Sample C1), 20–40 cm (Sample C4), 40–60 cm (Sample C2), 120–140 cm (Sample C3), and deeper than 280 cm (Sample C5). Among them, Sample C1 was collected by the Discovery remotely operated vehicle, sample C4 was collected by the television grab, and Samples C2, C3, and C5 were collected by the gravity sampler. Concentrations of sulfate and CH_4_ in surface sediments of sampling sites were respectively 28 mM and 2642 μM measured by Raman spectra and Hydro®CH_4_ (CONTROS) sensors. All sedimentary samples were dehydrated in an oven at 80°C until completely dry. After grinding, the sample powder was filtered through a 200‐mesh screen. The filtrate was analyzed for the chemical content of sulfur using an S8 Tiger X‐ray fluorescence spectrometer (BRUKER). These five cold seep sediment samples (C1, C4, C2, C3, and C5, 20 g each) were further used for metagenomic analysis in BGI. Briefly, total DNAs from these samples were extracted using the DNeasy® PowerSoil® Pro Kit (Qiagen) and the integrity of DNA was evaluated by gel electrophoresis. The libraries were prepared with 0.5 μg of each sample. DNA was sheared into fragments from 50 to 800 bp in length using a Covaris E220 ultrasonicator (Covaris); fragments between 150 and 250 bp were secreted using AMPure XP beads (Agencourt) and repaired using T4 DNA polymerase (ENZYMATICS). After ligated at both ends to T‐tailed adapters and amplified for eight cycles, the DNA fragments were used to produce single‐stranded, circular DNA libraries. All libraries were sequenced on a BGISEQ‐500 platform (BGI) to obtain 100 bp paired‐end raw reads. Quality control was performed via the program of SOAPnuke (v1.5.6) (setting: ‐l 20 ‐q 0.2 ‐n 0.05 ‐Q 2 ‐d ‐c 0 ‐5 0 ‐7 1)^64^. Gene prediction for metagenomics data was performed using Glimmer (v 3.02)^65^. And protein functions were annotated with Kyoto Encyclopedia of Genes and Genomes, Release 87.0, NR (Nonredundant Protein Database databases, 20180814), Swiss‐Prot (release‐2017_07), and EggNOG (2015‐10_4.5v) databases by default, and the best hits were chosen.

To understand the relative abundance of SRB belonging to *Desulfovibrionaceae* in deep‐sea cold seep, we selected these five cold seep sediment samples (C1, C4, C2, C3, and C5) for OTUs sequencing performed by Genesky (Genesky Biotechnologies Inc.). Total DNAs from sample C1 were extracted by FastDNA Spin Kit (MP), and 500 ng DNA was used as the PCR template. 16S rRNA fragments of distinct regions (V4/V5) were amplified using the specific primers with Illumina adapter sequences (515F: 5′‐GTGCCAGCMGCCGCGG‐3′ and 907R: 5′‐CCGTCAATTCMTTTRAGTTT‐3′). These PCR products were purified by a Gel Extraction Kit (Qiagen), and sequencing libraries were constructed using TruSeq® DNA PCR‐Free Sample Preparation Kit (Illumina) following the manufacturer's instructions. The library quality was detected with the Qubit 3.0 (Thermo Fisher Scientific) and Bioanalyzer 2100 system (Agilent). Then the library was sequenced on an Illumina MiSeq platform and 2 × 250 bp paired‐end reads were generated.

### Statistical analysis

The significant differences among groups were analyzed by the SPSS 18.0 program with one‐way analysis of variance and multiple comparisons. A statistical significance was defined in our study as *p* < 0.05 (indicated by * in all figures) or *p* < 0.01 (indicated by ** in all figures).

## AUTHOR CONTRIBUTIONS

Rui Liu and Chaomin Sun conceived and designed the study. Rui Liu conducted most of the experiments. Yeqi Shan helped to perform the in situ experiments. Shichuan Xi and Xin Zhang helped to perform the Raman spectra analysis. Rui Liu and Chaomin Sun lead the writing of the manuscript. All authors contributed to and reviewed the manuscript.

## ETHICS STATEMENT

This study has no animal or human experiments. There are no ethical issues involved.

## CONFLICT OF INTERESTS

The authors declare no conflict of interests.

## Supporting information

Supplementary information.

Supplementary information.

## Data Availability

The genomic data of *Oceanidesulfovibrio marinus* CS1 have been submitted to the NCBI with the accession number CP039543.1. The mass spectrometry proteomics data have been deposited to the Proteome Xchange Consortium via the PRIDE^66^ partner repository with the dataset identifier PXD023247. The metagenomic sequencing raw data have been deposited to NCBI Short Read Archive (accession numbers: SRR13052532, SRR13063401, SRR13336710, SRR13065122, and SRR13065132). The amplicon sequencing raw data have also been deposited to NCBI Short Read Archive (accession number: PRJNA675395).
