# Supplementary material for: A deep‐sea sulfate‐reducing bacterium generates zero‐valent sulfur via metabolizing thiosulfate
Source: mLife. 2022 Sep 23;1(3):257–71. doi: 10.1002/mlf2.12038 (PMC10989961; doi:10.1002/mlf2.12038)
Supplement: Supplementary file 2 — Supplementary information. [file MLF2-1-257-s002.pdf]

# A deep-sea sulfate-reducing bacterium generates zero-valent sulfur via metabolizing thiosulfate

Rui Liu[1,2,5], Yeqi Shan[1,2,3,5], Shichuan Xi[3,4,5], Xin Zhang[4,5], Chaomin Sun[1,2,5*]

[1]CAS and Shandong Province Key Laboratory of Experimental Marine Biology & Center of Deep Sea Research, Institute of Oceanology, Chinese Academy of Sciences, Qingdao, China

[2]Laboratory for Marine Biology and Biotechnology, Qingdao National Laboratory for Marine Science and Technology, Qingdao, China

[3]College of Earth Science, University of Chinese Academy of Sciences, Beijing, China

[4]CAS Key Laboratory of Marine Geology and Environment & Center of Deep Sea Research, Institute of Oceanology, Chinese Academy of Sciences, Qingdao, China

[5]Center of Ocean Mega-Science, Chinese Academy of Sciences, Qingdao, China

[*] Corresponding author

Chaomin Sun Tel.: +86 532 82898857; fax: +86 532 82898857.

E-mail address: sunchaomin@qdio.ac.cn

## Supplementary methods

**Proteomic profiling: Trypsin digestion, TMT labeling, HPLC fractionation, LC-MS/MS analysis of pepetides**

Briefly, the cells of strain CS1 were washed with 10 mM phosphate buffer solution (PBS pH 7.4), resuspended in lysis buffer (8 M urea, 1% Protease Inhibitor Cocktail) and disrupted by sonication. The remaining debris was removed by centrifugation at 12,000 *g* at 4 ℃ for 10 min. Finally, the supernatant was collected and the protein concentration was determined with a BCA protein assay kit (Thermo Fisher Scientific, USA) according to the manufacturer's instructions.

For digestion, the protein solution was reduced with 5 mM dithiothreitol for 30 min at 56 ℃ and alkylated with 11 mM iodoacetamide for 15 min at room temperature in darkness. The protein sample was then diluted by adding 100 mM TEAB to urea concentration less than 2M. Finally, trypsin was added at 1:50 trypsin-to-protein mass ratio for the first digestion overnight and 1:100 trypsin-to-protein mass ratio for a second 4 h-digestion. After trypsin digestion, peptide was desalted by Strata X C18 SPE column (Phenomenex) and vacuum-dried. Peptide was reconstituted in 0.5 M TEAB and processed according to the manufacturer's protocol for TMT kit. The tryptic peptides were fractionated into fractions by high pH reverse-phase HPLC using Agilent 300Extend C18 column (5 μm particles, 4.6 mm ID, 250 mm length). Briefly, peptides were first separated with a gradient of 8% to 32% acetonitrile (pH 9.0) over 60 min into 60 fractions. Then, the peptides were combined into 18 fractions and dried by vacuum centrifuging. The tryptic peptides were dissolved in 0.1% formic

acid (solvent A), directly loaded onto a home-made reversed-phase analytical column (15-cm length, 75 μm i.d.). The gradient was comprised of an increase from 6% to 23% solvent B (0.1% formic acid in 98% acetonitrile) over 26 min, 23% to 35% in 8 min and climbing to 80% in 3 min then holding at 80% for the last 3 min, all at a constant flow rate of 400 nL/min on an EASY-nLC 1000 UPLC system.

The peptides were subjected to NSI source followed by tandem mass spectrometry (MS/MS) in Q ExactiveTM Plus (Thermo) coupled online to the UPLC. The electrospray voltage applied was 2.0 kV. The m/z scan range was 350 to 1800 for full scan, and intact peptides were detected in the Orbitrap at a resolution of 70,000. Peptides were then selected for MS/MS using NCE setting as 28 and the fragments were detected in the Orbitrap at a resolution of 17,500. A data-dependent procedure that alternated between one MS scan followed by 20 MS/MS scans with 15.0s dynamic exclusion. Automatic gain control (AGC) was set at 5E4. Fixed first mass was set as 100 m/z. The concentration and total weight have been listed as follow:

| Group | Concentration (μg/μL) | Volume (μL) | Total weight (μg) |
|---|---|---|---|
| **Control** | 7.78 | 500 | 3888.3 |
| **$Na_2S_2O_3$** | 7.94 | 750 | 5957.7 |
| **$Na_2SO_3$** | 2.27 | 700 | 1586.6 |
| **$Na_2S$** | 8.00 | 950 | 7599.7 |
| **In situ** | 6.08 | 450 | 2736.6 |

**Proteome data analysis**

The resulting MS/MS data were processed using Maxquant search engine (v.1.5.2.8). Tandem mass spectra were searched against NCBI database concatenated with reverse decoy database. Trypsin/P was specified as cleavage enzyme allowing up to 2 missing

cleavages. The mass tolerance for precursor ions was set as 20 ppm in First search and 5 ppm in Main search, and the mass tolerance for fragment ions was set as 0.02 Da. Carbamidomethyl on Cys was specified as fixed modification and oxidation on Met was specified as variable modifications. FDR was adjusted to < 1% and minimum score for peptides was set > 40.

**The detection of thiosulfate**

This method uses 0.05 mM potassium dichromate ($K_2Cr_2O_7$) as a standard solution to titrate the remaining thiosulfate in the supernatant. Firstly, adding 200 μL $K_2Cr_2O_7$ standard solution, excess potassium iodide (KI), and 3 mL acid buffer (1:2 HCl) to the iodine flask. Secondly, the mixture is reacted for 3 min in the dark with shaking. Finally, adding 30 mL boiled distilled water and 100 μL 1% soluble starch (after activation) in the mixture. The initial position of the thiosulfate solution in the burette is recorded as volume 1 (V1). After the start of the titration, the color in the iodine measuring flask changes from dark blue to yellow-green to light blue, and the blue disappears at the end of the titration. Then the position of the thiosulfate solution in the burette is recorded as volume 2 (V2). The final volume of the titration is ΔV (V1-V2). Each sample is titrated three times as biological replicates.

**The detection of sulfate**

The concentration of sulfate in the supernatant was measured by a modified barium sulfate turbidimetry. Firstly, adding 120 μl barium chloride ($BaCl_2$) standard solution (0.14 M), 120 μL absolute ethanol, 40 μl Tween-80 (0.1 g/L) into a 2 mL centrifuge tube. Secondly, adding 0, 20, 40, 60, 80 and 100 μL sodium sulfate (20 mM) standard

solution, respectively, making up the volume to 1 mL with HCl solution (pH=1). After mixing well, taking 200 μL to measure the absorbance at 420 nm on a UV-Vis spectrometer (Infinite M1000 Pro; Tecan, Männedorf, Switzerland) and making a standard curve. Then the sulfate in sample is determined by the same system as the standard curve, and 50 μL of sample is added to the reaction. Each sample is measured three times as biological replicates.

**The detection of ZVS**

The ZVS ($S_8$) standard solution is gradually diluted with dichloromethane, and its absorbance value is measured at 270 nm (Infinite M1000 Pro; Tecan, Männedorf, Switzerland) and a standard curve is made. Totally, 1 mL of the bacterial medium is taken and placed in a glass test tube. Then 3 mL of dichloromethane is added in the tube with shaking for 15 s. After the reaction, the organic phase is carefully sucked by a pipette and transferred to a clean glass test tube. And more 2 mL of dichloromethane is added for the second extraction as above. Finally, the organic phase obtained from the two extractions is transferred to the same clean test tube and measured the absorbance at 270 nm. Each sample is measured three times as biological replicates.

**Table S1** Chemical parameters of sites for SRB isolation and *in situ* cultivation

| **Chemical parameters** | **The site for isolation (2017)** | **The site for *in situ* cultivation (2020)** |
| --- | --- | --- |
| Depth under the surface of ocean (m) | 1143 | 1146 |
| Longitude and latitude | 119 °17'04.956"E, 22 °06'58.384"N | 119 °17'04.429"E, 22 °07'01.523"N |
| pH | 7.68 | 7.59 |
| Temperature (℃) | 3.75 | 3.66 |
| $CH_4$ (μM) | 2169 | 1723 |
| Sulfate (mM) | 28.3 | 28.1 |
| Dissolved oxygen (mg/L) | 3.07 | 3.25 |
| $CO_2$ (PPM) | 812.34 | 815 |
| Salinity (‰) | 34.54 | 34.55 |

**Table S2** Composition of trace elements solution

| Components | Content |
|---|---|
| $Na_2EDTA \cdot 2H_2O$ (pH 8.0) | 29 g |
| $MnSO_4 \cdot H_2O$ | 3.26 g |
| $CoCl_2 \cdot 6H_2O$ | 1.8 g |
| $ZnSO_4 \cdot 7H_2O$ | 1.0 g |
| $NiSO_4 \cdot 6H_2O$ | 0.11 g |
| $GuSO_4 \cdot 5H_2O$ | 0.10 g |
| $H_3BO_3$ | 0.10 g |
| $KAl(SO4)_2 \cdot 12H_2O$ | 0.10 g |
| $Na_2MoO_4 \cdot 2H_2O$ | 0.10 g |
| $Na_2WO_4 \cdot 2H_2O$ | 0.10 g |
| $Na_2SeO_3$ | 0.05 g |
| Milli-Q water | 1 L |

**Table S3** Strains and plasmids and primers used in this study

| | Description or sequence | Notes |
|---|---|---|
| **Strains** | | |
| *Oceanidesulfovibrio marinus* CS1 | isolated from sediments of deep-sea cold seep | This study |
| *Escherichia coli* DH5α | $F^-$, φ80*lac*ZΔM15, Δ(*lac*ZYA-*arg*F)U169, *deo*R, *rec*A1, *end*A1, *hsd*R17($r_k^-$, $m_k^+$), *pho*A *sup*E44, $\lambda^-$, *thi*-1, *gyr*A96, *rel*A1 | Commercial |
| *E*. *coli* BL21(DE3) | $F^-$, *omp*T, *hsd*$S_B$($r_B^-$, $m_B^-$), *gal*, *dcm*(DE3) | Commercial |
| **Plasmids** | | |
| pMD19-T simple | High copy number cloning vector, [a]$Amp^r$ | Commercial |
| pET28a (+) | Prokaryotic expression vector, [a]$Kan^r$ | Commercial |
| pET28a (+)/*sqr* | pET28a (+) containing the ORF[b] of *sqr* gene, $Kan^r$ | This study |
| pET28a (+)/*phsA* | pET28a (+) containing the ORF of *phsA* gene, $Kan^r$ | This study |
| pET28a (+)/*sqr-phsA* | pET28a (+) containing both ORFs of *sqr* gene and *phsA* gene, $Kan^r$ | This study |
| **Primers** | | |
| sqr-F | CG<u>GGATCC</u>ATGCGCAAGATTCTGATCATCGG | *BamH*I |
| sqr-R | CC<u>GAATTC</u>AGGCTTTCCAGCGGCAAGTCCA | *EcoR*I |
| phsA-F | CG<u>GAATTC</u>ATGAGCGATTTCAAAACTGTCTACA | *EcoR*I |
| phsA-R | CGG<u>CTCGAG</u>CGTCTTGCGCACTTCGATG | *Xho*I |
| sqr-RTF | TGCTGGTCTCCATCCCG | |

| | |
|---|---|
| sqr-RTR | CCTGTGCCTTGAGCGTGT |
| phsA-RTF | CCGACAAGTGGATTCAGGAC |
| phsA-RTR | TGGATGCCAGATCACAGACG |
| 16S-RTF | CCCAATCACCAGCCCTACC |
| 16S-RTR | GGTCACGCCAATCCCAAA |

[a] $Amp^r$, ampicillin resistance; $Kan^r$, kanamycin resistance.

[b] ORF, open reading frame.

**Table S4** Chemical parameters of the sampling sites for metagenomic sequencing and amplicon analysis

| **Chemical parameters for sample site** | **C1** | **C4** | **C2** | **C3** | **C5** |
|---|---|---|---|---|---|
| Depth under the surface of ocean (m) | 1139 | 1121 | 1137 | 1137 | 1137 |
| Depth under the surface of sediment (cm) | 0~20 | 20~40 | 40~60 | 120~140 | >280 |
| Longitude and latitude | E 119 °17'09.106"<br>N 22 °06'55.114" | E 119 °17'07.322"<br>N 22 °06'58.598" | E 119 °17'06.436"<br>N 22 °06'55.169" | E 119 °17'06.436"<br>N 22 °06'55.169" | E 119 °17'06.436"<br>N 22 °06'55.169" |
| pH | 7.62 | NA | NA | NA | NA |
| Temperature ( ℃) | 3.64 | NA | NA | NA | NA |
| Salinity (‰) | 34.49 | NA | NA | NA | NA |
| Dissolved oxygen (mg/L) | 3.03 | NA | NA | NA | NA |
| $CO_2$ (PPM) | 828 | NA | NA | NA | NA |
| $CH_4$ (μM) | 2642 | NA | NA | NA | NA |
| Sulfate (mM) | 28 | NA | NA | NA | NA |
| S (PPM) | 3452.3 | 3644.6 | 5264.1 | 1860.1 | 3857.5 |

NA, not available.

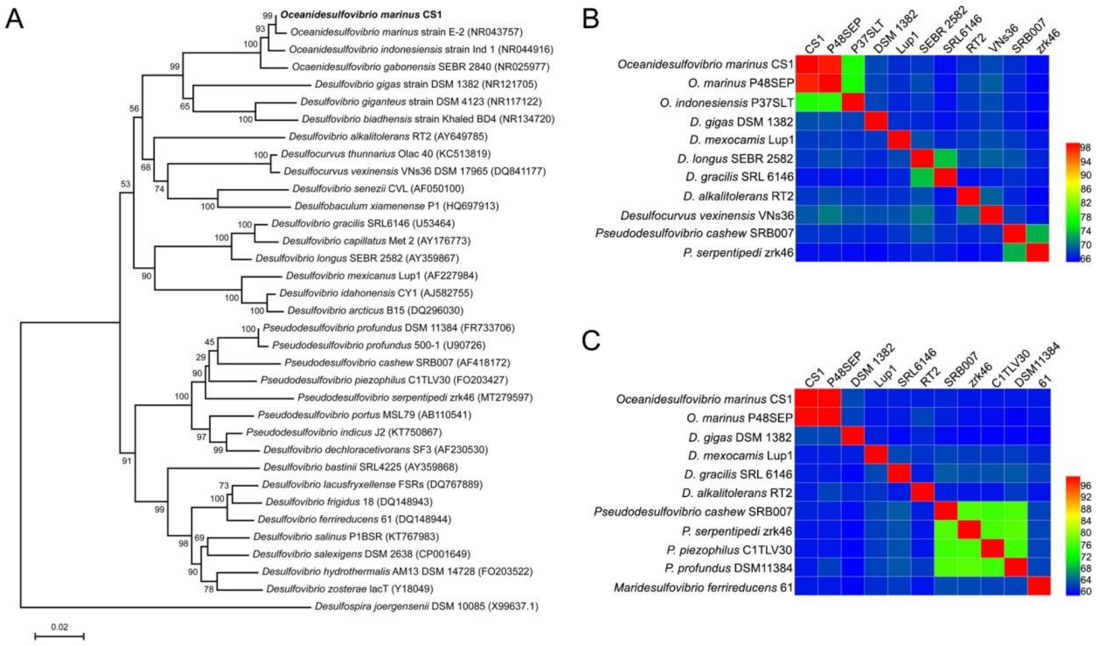


**Fig. S1.** Phylogenetic analysis of *O. marinus* CS1. (A) Phylogenetic placement of strain CS1 within the representative species in the family *Desulfovirbionaceae* based on almost complete 16S rRNA gene sequences. The tree is inferred and reconstructed under the the neighbor-joining criterion and expressed as percentages of 1,000 replications. Bar, 0.02 substitutions per nucleotide position. (B-C) The ANI and AAI analyses of genomes between strain CS1 and other strains in the family *Desulfovirbionaceae*, respectively. The accepted threshold of ANI and AAI for same species is above 95%.

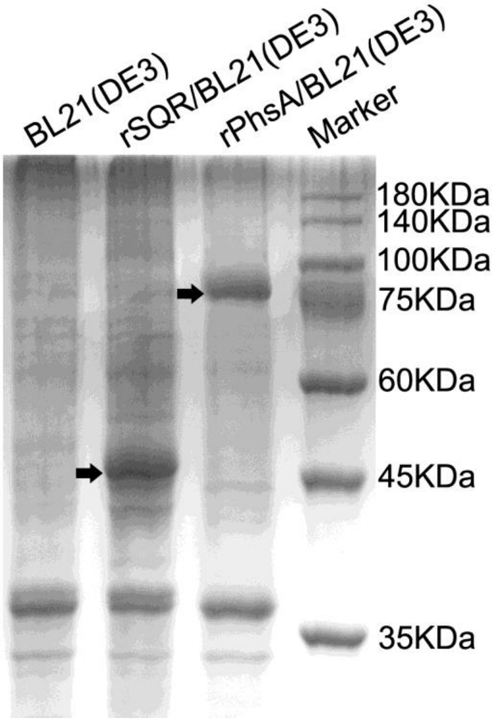


**Fig. S2.** Detection of the overexpression of SQR and PhsA in *E. coli* BL21(DE3) via SDS-PAGE. Line 1, BL21(DE3) transformed with pET28a(+) as the negative control. Line 2, SQR of *O. marinus* CS1 was overexpressed in *E. coli* BL21(DE3). The arrow indicates the position of His-tagged SQR in the supernatant of *E. coli sqr/*pET28a (+)/BL21(DE3). Line 3, PhsA of *O. marinus* CS1 was overexpressed in *E. coli* BL21(DE3). The arrow indicates the position of His-tagged PhsA in the supernatant of *E. coli phsA/*pET28a (+)/BL21(DE3). Line 4, Standard molecular weight protein marker.

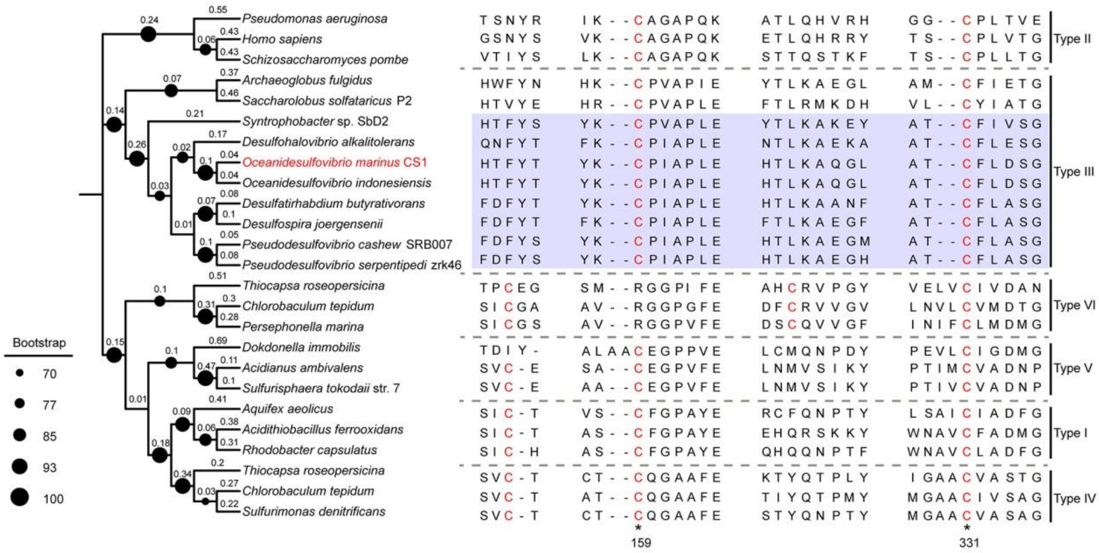


**Fig. S3.** Phylogenetic analysis and sequence alignment of SQR homologs identified in *O. marinus* CS1 and other bacteria. The conserved sites of cysteine in different SQRs are highlighted with red color. The numbers at the bottom represent positions of two conserved cysteines in the SQR identified in *O. marinus* CS1. The tree is inferred and reconstructed under the maximum likelihood criterion, and bootstrap values (%) > 70 are indicated at the base of each node (expressed as black nots with different sizes). Percentages represent the tree scale of each branch.

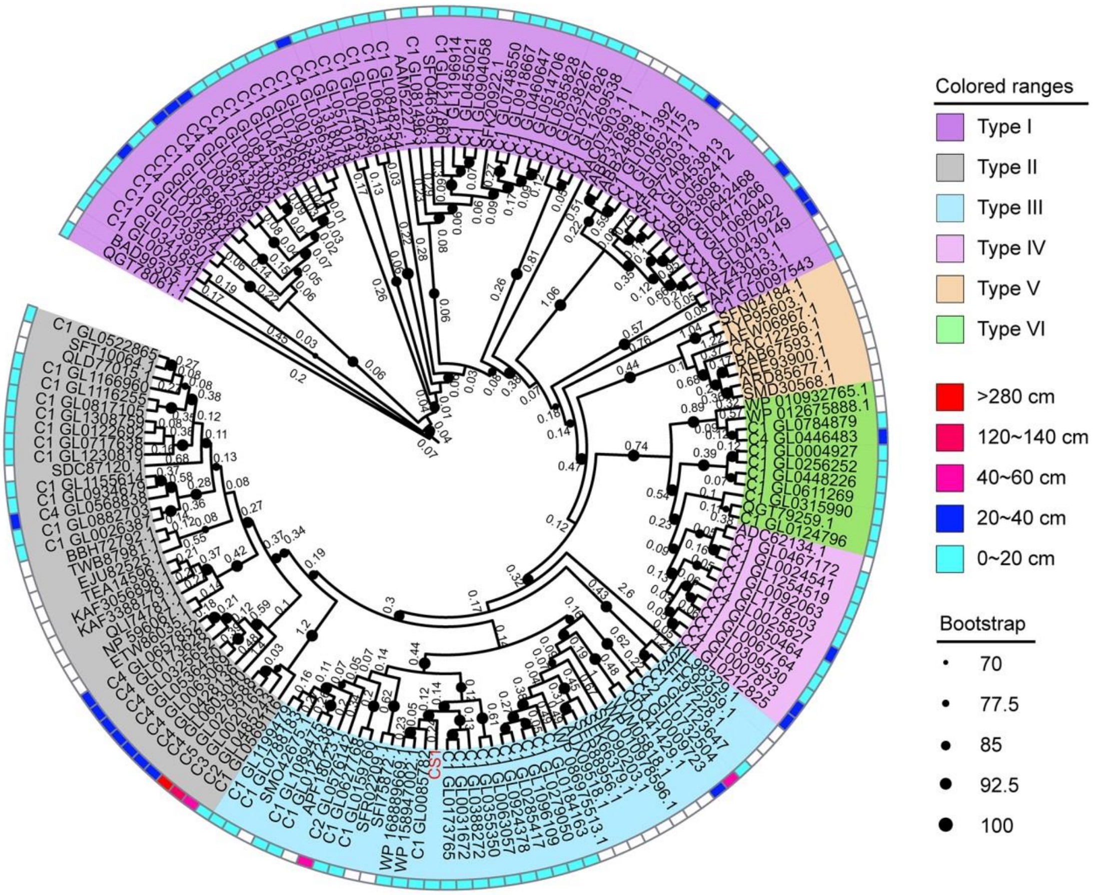


**Fig. S4.** Maximum-likelihood phylogeny of sulfide:quinone oxidoreductases (SQRs) identified in metagenomic data derived from deep-sea cold seep sediments (1,000 bootstrap replicates). Bootstrap values (%) > 70 are indicated at the base of each node (expressed as black nots with different sizes). Percentages represent the tree scale of each branch. The detailed information of proteins used for phylogenetic analyses is listed in the Supplementary Dataset Sheet 5.
